# A systematically biosynthetic investigation of lactic acid bacteria reveals diverse antagonistic bacteriocins that potentially shape the human microbiome

**DOI:** 10.1186/s40168-023-01540-y

**Published:** 2023-04-27

**Authors:** Dengwei Zhang, Jian Zhang, Shanthini Kalimuthu, Jing Liu, Zhi-Man Song, Bei-bei He, Peiyan Cai, Zheng Zhong, Chenchen Feng, Prasanna Neelakantan, Yong-Xin Li

**Affiliations:** 1 Department of Chemistry and The Swire Institute of Marine Science, The University of Hong Kong, Pokfulam Road, Hong Kong, China; 2 The University of Hong Kong Shenzhen Institute of Research and Innovation, Shenzhen, China; 3 Division of Restorative Dental Sciences, Faculty of Dentistry, The University of Hong Kong, Hong Kong, China; 4 Department of Urology, Huashan Hospital, Fudan University, Shanghai, 200040 China

**Keywords:** Lactic acid bacteria, Biosynthetic gene clusters, Secondary metabolites, Bacteriocins, Human microbiome, Vaginal microbiome

## Abstract

**Background:**

Lactic acid bacteria (LAB) produce various bioactive secondary metabolites (SMs), which endow LAB with a protective role for the host. However, the biosynthetic potentials of LAB-derived SMs remain elusive, particularly in their diversity, abundance, and distribution in the human microbiome. Thus, it is still unknown to what extent LAB-derived SMs are involved in microbiome homeostasis.

**Results:**

Here, we systematically investigate the biosynthetic potential of LAB from 31,977 LAB genomes, identifying 130,051 secondary metabolite biosynthetic gene clusters (BGCs) of 2,849 gene cluster families (GCFs). Most of these GCFs are species-specific or even strain-specific and uncharacterized yet. Analyzing 748 human-associated metagenomes, we gain an insight into the profile of LAB BGCs, which are highly diverse and niche-specific in the human microbiome. We discover that most LAB BGCs may encode bacteriocins with pervasive antagonistic activities predicted by machine learning models, potentially playing protective roles in the human microbiome. Class II bacteriocins, one of the most abundant and diverse LAB SMs, are particularly enriched and predominant in the vaginal microbiome. We utilized metagenomic and metatranscriptomic analyses to guide our discovery of functional class II bacteriocins. Our findings suggest that these antibacterial bacteriocins have the potential to regulate microbial communities in the vagina, thereby contributing to the maintenance of microbiome homeostasis.

**Conclusions:**

Our study systematically investigates LAB biosynthetic potential and their profiles in the human microbiome, linking them to the antagonistic contributions to microbiome homeostasis via omics analysis. These discoveries of the diverse and prevalent antagonistic SMs are expected to stimulate the mechanism study of LAB’s protective roles for the microbiome and host, highlighting the potential of LAB and their bacteriocins as therapeutic alternatives.

Video Abstract

**Supplementary Information:**

The online version contains supplementary material available at 10.1186/s40168-023-01540-y.

## Introduction

Lactic acid bacteria (LAB) are Gram-positive, microaerophilic bacteria, which have drawn extensive attention due to their fundamental roles in different biological processes [1]. LAB are known for their ability to produce lactic acid during carbohydrate metabolism, which is essential in food fermentation and has led to their widespread use in the food industry [2]. Due to their inherent safety, LAB have been genetically engineered to produce food additives, drugs, and therapeutic molecules [1–4]. More importantly, a growing body of evidence reveals that certain LAB strains can confer significant health benefits when consumed, making them attractive candidates for probiotics [5]. LAB may exert their probiotic effects through multiple mechanisms, including regulating gut microflora, producing bioactive metabolites, and modulating the immune system, all of which endow LAB with a protective role for the host [6]. Despite numerous studies focusing on characterizing LAB as probiotics for microflora regulation, how they impact microbiome homeostasis and host physiology is still not fully understood.

Metabolic crosstalk plays a fundamental role in how microbes, including LAB, interact with the host and maintain microbiome homeostasis. One way these interactions occur is through the production of a multitude of metabolites by the microbes, including secondary metabolites (SMs) such as antibiotics and pigments. SM-mediated interactions, such as mutualism and antagonism, are crucial in maintaining microbiome homeostasis [7, 8]. In particular, many LAB members, including *Lactobacillus, Streptococcus*, and *Lactococcus*, produce a diverse range of bioactive SMs, ranging from bacteriocins like nisin and lactocillin to tetramic acid reutericyclin [9–11]. Bacteriocins are ribosomally synthesized antimicrobial peptides with antibacterial potential and are generally divided into three classes [class I, post‐translationally modified peptides (e.g., nisin and lactocillin); class II, small unmodified peptides (e.g., amylovorin L and crispacin A); class III, large, heat-labile peptides] [12, 13]. Bacteriocins, which are the most extensively studied secondary metabolites produced by LAB, have been shown to modulate the microbial composition and inhibit pathogens, suggesting their potential role in shaping the microbiota [14]. However, previous studies on LAB secondary metabolites have mainly focused on structures, biosynthesis, mechanisms of action, or therapeutic potential in preventing infection on a case-by-case basis [15]. Therefore, the landscape of LAB SMs, particularly their diversity, prevalence, and potential roles in the human microbiome remains elusive, making it challenging to determine their active involvement in microbiome homeostasis.

With the development of bioinformatics techniques, the recent explosion of sequenced bacterial genomes and metagenomes provides fresh opportunities for large-scale biosynthetic analysis at both single species and community levels. Here we harnessed recent advances in biosynthetic and metagenomic analysis to investigate the untapped biosynthetic potential of LAB SMs systematically. Leveraging this comprehensive analysis of 31,977 LAB genomes and 748 human microbiome metagenomes, we gained previously undescribed insights into the biosynthetic capacity of LAB SMs and their diversity, abundance, and distribution in the human microbiome. Our study reveals that most secondary metabolite biosynthetic gene clusters (BGCs) may encode antagonistic SMs uncharacterized yet. We discovered a new class II bacteriocin, named crispacin 467, which may play a protective role in the human microbiome. To our best knowledge, this is the first largest survey of LAB biosynthetic potential and their profile in the human microbiome, linking them to the antagonistic contributions to microbiome homeostasis via omics analysis. Our omics-guided findings of the diverse and prevalent antagonistic bacteriocins in the human microbiome, particularly the vaginal microbiome, provide insight into antagonistic interactions linked to microbiome homeostasis and highlight the probiotic potential of LAB with antagonistic SMs.

## Results

### Genomic analysis reveals the landscape of SM biosynthetic potential of LAB

**Fig. 1:**
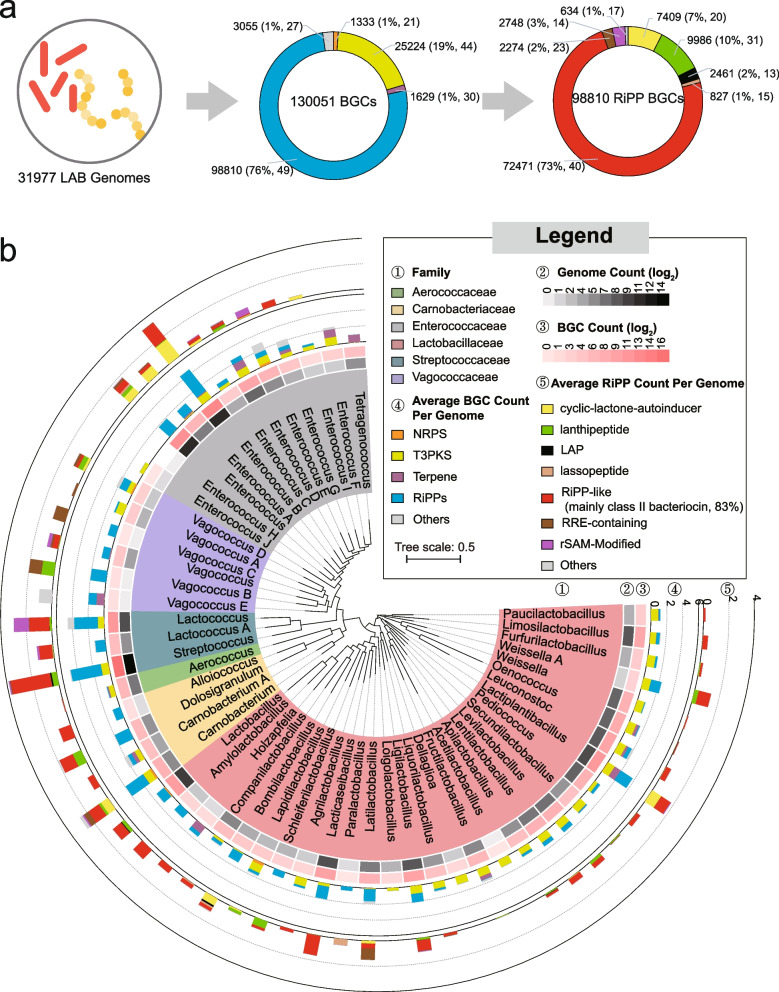
Overview of secondary metabolite biosynthetic capacity in LAB. **a** Overall BGCs identified from 31,977 LAB genomes. The numbers outside the brackets indicate BGC count, and numbers inside represent the corresponding percentage and the count of genera in which BGCs are present. The donut chart in the center displays the overall class distribution of BGCs, with corresponding colors matching the fourth layer of (b). Meanwhile, the donut chart on the right exhibits the class distribution of RiPP BGCs, with colors corresponding to the fifth layer in (b). **b** Layers are as follows: ①, the maximum likelihood phylogenetic tree based on 120 concatenate marker genes of 56 representative LAB genomes. LAB in this study covers 6 families and 56 genera under GTDB taxonomy; ②, the count of genomes included in this study, with being transformed with log2; ③, log2-transformed BGC count; ④, average BGC abundance in LAB genera; ⑤, average RiPPs abundance in LAB genera. In RiPPs terms, other subtypes of BGCs and hybrid BGCs are clustered into “Others”. rSAM-Modified RiPPs consist of RaS-RiPP, ranthipeptide and sactipeptide

Given that environment or foods are possible LAB sources for the gut microbiome, to profile LAB SMs in the human microbiome, we first gathered LAB genomes from different sources to comprehensively investigate the biosynthetic potential of LAB SMs. Publicly available bacterial single amplified genomes (SAGs) and metagenome-assembled genomes (MAGs) of LAB were gathered from three databases (RefSeq [16], PATRIC [17], and IMG/M [18]) and two previous studies [19, 20], resulting in 40,879 SAGs and 4,575 MAGs in total (Supplementary Table 1). Genomes were then de-duplicated, and their taxonomical classifications were verified and unified using GTDB taxonomy. As a result, 31,977 LAB genomes (27,549 SAGs and 4,428 MAGs, Supplementary Table 2), spanning six families containing 56 genera, were retained for global biosynthetic analysis of LAB SMs (Supplementary Fig. 1a). Using a rule-based BGC detection tool, antiSMASH 6.0 [21], we identified 130,051 BGCs from 30,718 genomes (Fig. 1a, Supplementary Fig. 1b, Supplementary Table 3), including 1,333 nonribosomal peptide synthetase clusters (NRPS, 1.0%), 25,278 polyketides synthase clusters (PKS, 19.7%), 98,810 ribosomally encoded and post-translationally modified peptides (RiPPs, 76.0%), 1,629 terpene (1.3%) and 2,984 BGCs (2.3%) encoding other types of metabolites (Supplementary Fig. 2). The BGCs per genome ranged from 0 to 14, with an average of 4.07. Among the most abundant RiPPs, RiPP-like (formerly annotated as bacteriocin by antiSMASH) topped its list with 72,471 (55.7% of total BGCs). Using BiG-SLiCE [22] to extract bacteriocin biosynthesis-related domains from 72,471 RiPP-like BGCs (Supplementary Fig. 3 and Supplementary Table 4), we identified 60,497 class II bacteriocins (RiPP-like BGCs that contain class II bacteriocins-related domains identified by BiG-SLiCE) (46.5%), which are the most abundant LAB SMs (Fig. 1a).

To gain insight into the phylogenetic distribution of BGC in LAB genera, we examined 26,983 BGC-containing SAGs, excluding MAGs due to their incompleteness. The biosynthetic capacity varied considerably at the family, genus, or species level (Fig. 1b, Supplementary Fig. 4). The RiPPs and T3PKS BGCs dominated all LAB genera except for *Tetragenococcus*, while terpene BGCs and NRPS BGCs were sporadically distributed in those genera (Fig. 1, Supplementary Figs. 5 and 6). Among 55 genera, we found a median of ≥ 1 RiPPs per genome in 28 genera and one T3PKS in 33 genera. While a high proportion of RiPPs has been reported in Firmicutes [23], T3PKS dominating in certain LAB genera might encode specialized metabolites that have basic biological functions. Of note, despite being small in genome size, Streptococcaceae generally harbored more abundant BGCs than other families (Supplementary Fig. 4a), with a median of five BGC per genome, exemplified by genera *Lactococcus* and *Streptococcus*. Contrastingly, 33 genera only harbored a median of ≤ 1 BGC per genome, indicating the limited biosynthetic capacity of the LAB majority (Fig. 1b). By comparing 53 LAB genera and 3,805 non-LAB genera (164,417 genomes, Supplementary Table 5), we found comparatively limited biosynthetic capacity in LAB and a significantly strong correlation (Spearman *rho* = 0.712, *P* < 0.001) between bacterial biosynthetic potential and their genome size (Supplementary Fig. 7). The small genomes and reduced biosynthetic capacities in LAB might correlate with their adaptation to nutritionally-rich niches.

### Most LAB BGCs are species-specific and uncharacterized yet

**Fig. 2:**
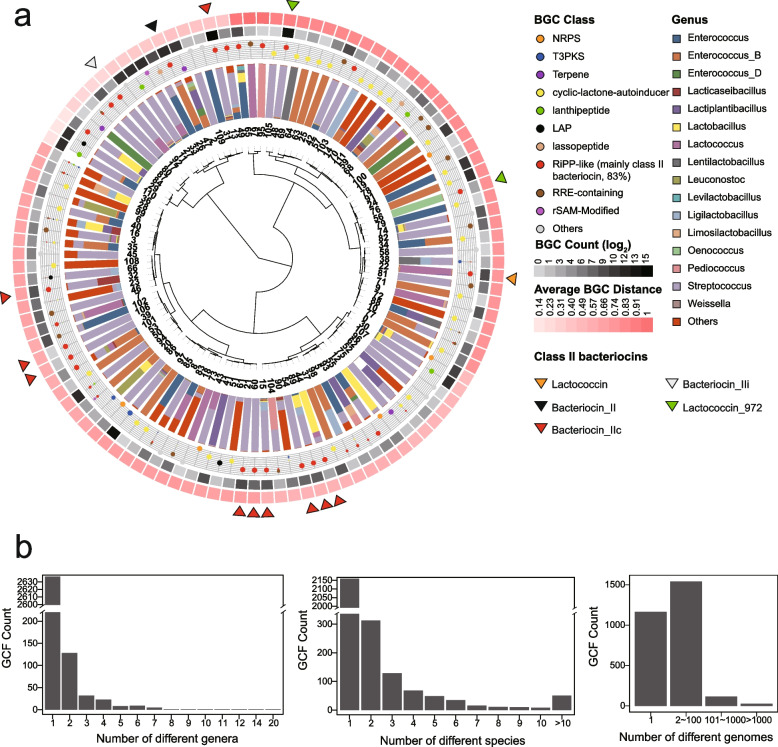
LAB BGCs are diverse and taxa-specific. **a** The figure shows the clustering of 129,878 biosynthetic gene clusters (BGCs) into 2,849 gene cluster families (GCFs) and 112 gene cluster clans (GCCs). The innermost dendrogram displays the hierarchical clustering of 112 GCCs based on their average cosine distance to MIBiG BGCs. The next outer layer illustrates the proportion of different genera, with genera having less than 200 genomes grouped into "Others". The subsequent layer represents the proportion of BGC classes, with the size of the points being proportionate to their representation. The two outer layers refer to log2-transformed BGC count and average distance to MiBiG BGCs. The triangles denote clans dominated by particular class II bacteriocins-related domains (proportion > 80% in one clan). The predominant bacteriocins-related domains are shown in Supplementary Fig. 10. **b** The bar plot shows the number of GCFs present in different genera (left), species (medial), and genomes (right)

Although BGCs highly vary in gene content, grouping them into families (GCFs) or clans (GCCs) based on architectural relationships of biosynthetic elements is an effective way to uncover the similarity of their encoding products in terms of the chemical features and biological functions [24]. To gain insight into the novelty and diversity of 130,051 LAB BGCs, we extracted BGC features (biosynthetic domains) using BiG-SLiCE [22] and grouped them based on an all-to-all cosine distance among BGCs [25]. The 129,878 BGCs with features were classified into 2,849 GCFs and 112 GCCs, with a distance threshold of 0.2 and 0.8, respectively (Fig. 2, Supplementary Fig. 8). We further compared the 112 GCCs to the reference known BGCs described in the 'Minimum Information about a Biosynthetic Gene' (MIBiG) repository [26]. Our analysis revealed that only three clans, namely linear azol(in)e-containing peptides (LAP, GCC_29), RiPP-like (GCC_84), and class II lanthipeptides (GCC_110), were found to be closely similar to known BGCs, with an average cosine distance of less than 0.2. This finding underscores the significant knowledge gap in LAB secondary metabolites and highlights the vast potential for discovering novel chemistry from these bacteria. Of note, the majority of NRPS (73.2%), terpene (99.3%) and T3PKS (99.3%) were clustered into one respective clan. In contrast, RiPP BGCs, contributing 83 GCCs with 1,818 GCFs (RiPP proportion > 80% in GCCs or GCFs), were highly diverse due to the diversity of their post-translational modification (PTM) enzyme genes and adjacent genes (Fig. 2a). Among them, 621 GCFs of 23 RiPP-like GCCs encoded class II bacteriocins, representing one of the most diverse LAB SMs. The other 60 RiPP GCCs mainly encoded class I bacteriocins, including lanthipeptide, LAP, lassopeptide, and rSAM-modified RiPPs.

In prokaryotic genome evolution, the conserved genes across genera are more likely to contribute to essential ecological processes, whereas species- or even strain-specific genes often arise from natural selection, thus enhancing niche adaptation or host fitness [27]. In this context, we next examined the distribution and diversity of genus- or species-specific BGCs. While GCC clustering shows the distribution and novelty of LAB SMs, a fine resolution of GCF clustering can offer an insight into the diversity of BGCs that are predicted to encode similar natural products [28]. We found that the majority of GCFs were genus-specific (92.6%, 2,637/2,849) and species-specific (75.8%, 2,159/2,849). Remarkably, 1,165 GCFs (40.9%) contained only one BGC harbored by a specific strain (Fig. 2b). In contrast, only 7% (212) were cross-genus GCFs, including 142 RiPPs (present in 2–20 genera), 17 NRPS (in 2–3 genera), 21 T3PKS (in 2–9 genera), and 9 terpenes (in 2–7 genera) (Supplementary Figs. 9 and 10). Among these 142 cross-genus RiPP GCFs, 62 were class II bacteriocins. Owing to this high GCF diversity between genera, we did not observe a phylogenetic relationship in GCF presence/absence (Supplementary Fig. 8). These taxa-specific BGCs usually encode specialized SMs and provide a competitive edge to the producer for niche adaption. Considering the wide presence of LAB in different niches [29], a high proportion of species- and strain-specific BGCs might result from niche selection.

### LAB BGCs are diverse and niche-specific in the human microbiome

**Fig. 3:**
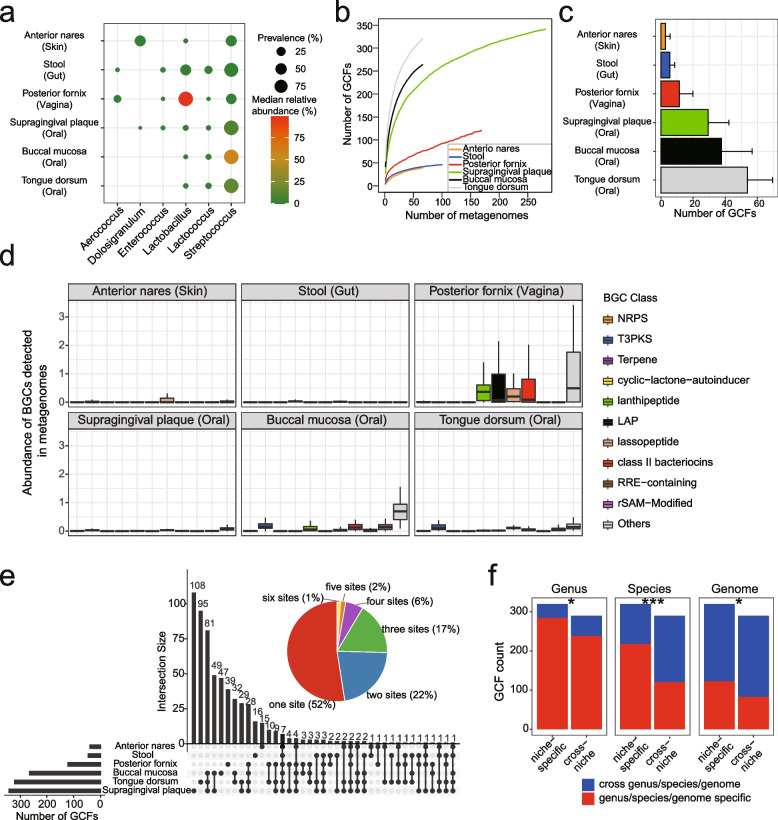
LAB BGCs in the human microbiome are variable and niche-specific. **a** The prevalence and abundance of LAB genera in the microbial communities of six body sites. The point size and color are proportionate to the genus prevalence and median abundance, respectively. **b** The GCF accumulation curve shows how the number of detected GCFs increases as more samples are included. The numbers of metagenomes of six body sites are as follows: anterior nares, 66; stool, 100; posterior fornix, 169; supragingival plaque, 281; buccal mucosa, 66; tongue dorsum, 66. **c** Number of GCFs detected in six body sites. Data are mean ± standard deviation, with only the upper error bar being shown. The color is indicated in the legend of (b). **d** Boxplot shows the abundance of different BGCs detected in six body sites. **e** The pie chart shows the proportion of 610 GCFs detected in how many sites and is distinguished using different colors. Corresponding percentages are shown in the brackets. The bar plot on the left refers to the number of GCFs in each site. The bar plot on the top depicts the number of GCFs of each intersection. Connecting lines are drawn if an intersection is present in more than one site. **f** Numbers of genus/species/genome-specific GCFs and cross-genus/species/genome GCFs that were detected in one site (niche-specific) or more than one sites (cross-niche). The Chi-squared test gave *P* values. *, 0.01 < *P* < 0.05; ***, *P* < 0.001

Numerous studies have revealed the variable prevalence of LAB species in the human microbiome [20], raising the question of to what extent their SMs vary in different body sites for niche adaption. Thus, we next explored the profile of LAB BGCs in the healthy human microbiome by re-visiting 748 metagenomes of six body sites from the Human Microbiome Project (HMP) [30]. These sites included aerobic (anterior nares, representing skin microbiome), microaerobic (supragingival plaque, buccal mucosa, tongue dorsum, and posterior fornix, representing oral and vaginal microbiome), and anaerobic (stool, representing gut microbiome) environments (Supplementary Table 6). In line with a previous larger-scale study [20], LAB exhibited variable abundance and prevalence in different body sites (Fig. 3a). Of note, the genus *Lactobacillus* dominated in the vagina with a median abundance of 99.0% [interquartile range (IQR), 91.0%-99.8%], whereas *Streptococcus* was moderately abundant but highly prevalent in six body sites.

To profile the diversity, abundance, and distribution of LAB BGCs in the human microbiome, we de-duplicated 130,051 BGCs to 24,222 representative BGCs and mapped metagenomic reads to 24,222 nonredundant BGCs. The number of LAB BGCs detected in six body sites varied considerably, with the highest in the oral cavity and the lowest in the skin (Supplementary Fig. 11a), probably due to the variable abundance of LAB. From 748 metagenomes, we detected 5,687 BGCs of 610 GCFs, including 71 T3PKS and 312 RiPPs with 92 class II bacteriocins (Supplementary Fig. 11b). The GCF accumulation curve indicated that more GCFs would be detected in those body sites as more samples were included, revealing the huge diversity of LAB SMs in the human microbiome (Fig. 3b). The three oral sites were the richest in GCFs (averaging 29, 38, and 54). Compared to the oral cavity, the vagina harbors a lower diversity of GCFs (averaging 12) but a significantly higher abundance of LAB BGCs (Figs. 3c, d). Particularly, the vaginal microbiome harbored a high abundance of class II bacteriocins, lassopeptide, lanthipeptide, and LAP. Of note, influenced by sequencing depth, the diversity and abundance of LAB BGCs in the human microbiome may be underestimated. Of 610 detected GCFs, ~ 52% were niche-specific in one of six sites, which accounted for 18%—38% of GCFs in a particular site (Fig. 3e). We also observed that those niche-specific GCFs were generally species-specific (Chi-squared test, *P* < 0.001), but not genus-specific (*P* = 0.026) nor strain-specific (*P* = 0.013) (Fig. 3f). This result indicated that niche-specific GCFs were derived from different species residing in distinct niches, which may provide a competitive advantage to the niche adaptation of their hosts. Our genomic and metagenomic analysis of biosynthetic potential revealed that the LAB SMs are diverse and variably prevalent in the human microbiome.

### Machine learning models reveal that most BGCs may encode antagonistic SMs

**Fig. 4:**
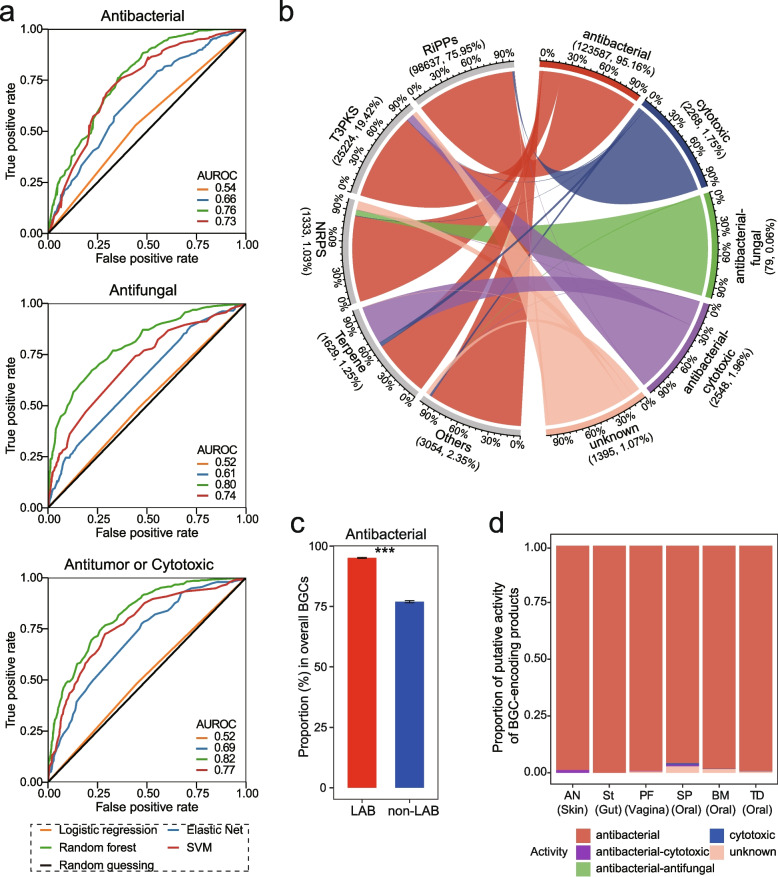
Putative compound activity of LAB BGCs. **a** Performance of four machine learning classifiers [logistic regression, elastic net regression, support vector machines (SVM), and random forest] in determining compound activities using tenfold cross-validation. The receiver operating characteristic (ROC) curves were based on aggregated performances of tenfold cross-validation. Average AUROC was shown. **b** Chord diagram showing the predicted activity of 129,878 BGCs. The scale was the proportion of each BGC class or predicted activity. The number shown in brackets refers to the BGC count and percentage relative to overall BGCs. Antibacterial-antifungal and antibacterial-cytotoxic represent BGCs encoding bifunctional SMs. The connections between BGCs and their predicted activities are highlighted with different colors according to the activity types. **c** Proportion of antibacterial SMs encoded by BGCs from LAB and non-LAB species. The proportion of antibacterial activity was calculated from randomly selected 10,000 BGCs of LAB or non-LAB, with be resampled 1,000 times. Data are mean ± standard deviation. *P* value was given by Wilcoxon rank-sum test (two sided), with “***” denoting *P* < 0.001. **d** The proportion of putative activities of BGCs detected in six body sites. AN, anterior nares; St, stool; PF, posterior fornix; SP, supragingival plaque; BM, buccal mucosa; TD, tongue dorsum

Given the abundance and prevalence of LAB BGCs in the human microbiome, we next want to study the potential bioactivities of BGC-encoding SMs. The bioactivity of SMs encoded by BGCs was recently predicted using machine learning strategies based on chemical fingerprints of predicted compound structure, protein family (PFAM) domains, and other genetic features [31–33]. Here, we adapted four common machine learning classifiers (logistic regression, elastic net regression, random forest, and support vector machines) to predict the bioactivities of LAB-derived SMs. For the training data (950 known BGCs, Supplementary Table 7, Supplementary Fig. 12), ten-fold cross-validation revealed that the random forest classifier outperformed others with an average area under the receiver operating characteristic curve (AUROC) being 0.76, 0.80, and 0.82, for antibacterial, antifungal, and antitumor or cytotoxic, respectively (Fig. 4a, Supplementary Fig. 13). The performance of the random forest classifier was comparable with previously reported methods [31, 32].

Using the random forest classifier, 129,878 LAB BGCs with features were predicted to encode different bioactive SMs, comprising antibacterial (*n* = 123,587, 95.2%), cytotoxic (*n* = 2,268, 1.8%), antibacterial-antifungal (*n* = 79, 0.1%), antibacterial-cytotoxic (*n* = 2,548, 2.0%), unknown (*n* = 1,395, 1.1%). Most BGCs, regardless of BGC classes, were predicted to be antibacterial (Fig. 4b). Of note, 97.8% of RiPPs (96,466/98,637) were predicted to exhibit antibacterial activity, implying that bacteriocins were plenteous in LAB. With predicted antibacterial activity, most RiPPs with known post-modifications were classified as class I bacteriocins, while most RiPPs-like (83.4%) were class II bacteriocins. Almost 100% of class II bacteriocins (60,494/60,497) were captured as antibacterial SMs, contributing to 48.1% of LAB-derived antibacterials. Those antibacterial BGCs dominated almost all LAB genera (Supplementary Fig. 14), possibly conferring a competitive edge in the microbial community. Compared to BGCs (*n* = 1,121,156) identified from non-LAB genomes, LAB-derived BGCs potentially encoded a significantly higher proportion of antibacterial SMs, indicating a higher antagonistic potential of LAB SMs (Wilcoxon rank-sum test, *P* < 0.001) (Fig. 4c, Supplementary Fig. 15a, b). A low percentage of LAB-derived BGCs encoding putative cytotoxic or antifungal SMs were found in specific species (Supplementary Fig. 15c, d). For example, a certain family of LAP (GCF_199) possibly conferring cytotoxic activity was distributed in ten *Streptococcus* species, especially *Streptococcus pyogenes*, in which common pathogenicity feature endowed by conserved LAP had been reported [34]. In the six body sites, most detected BGCs were predicted to encode antimicrobials (Fig. 4d), suggesting a potential role in bacterial antagonism for maintaining microbiome homeostasis. It is plausible that LAB containing such antimicrobial SMs, particularly class II bacteriocins, may provide protective benefits to the host against pathogen invasion and contribute to microbiome homeostasis through antagonistic interactions [7].

### Underexplored class II bacteriocins are widely distributed in the human microbiome

**Fig. 5:**
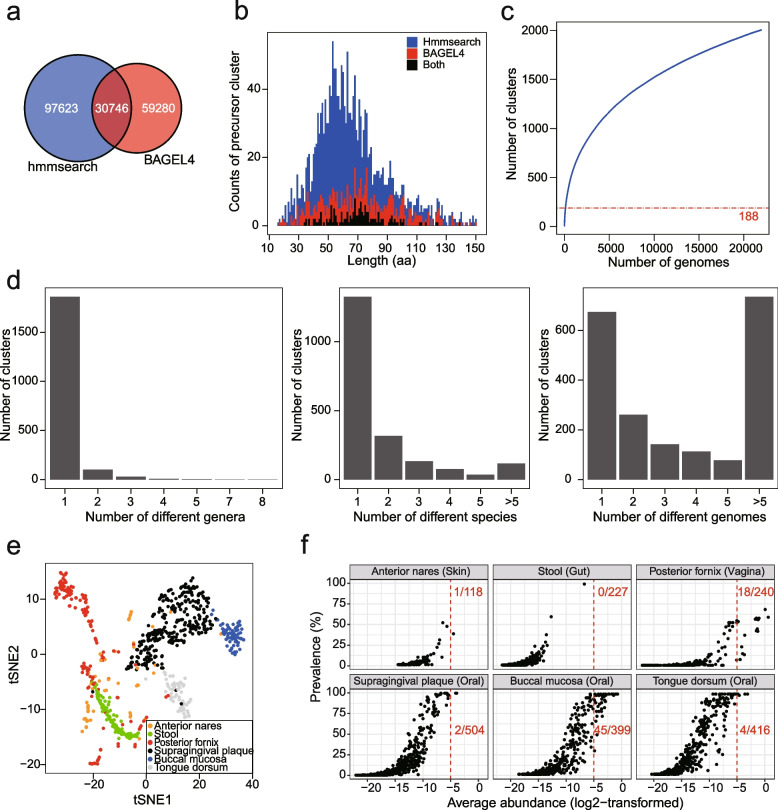
Class II bacteriocins are structurally diverse and variably prevalent in the human microbiome. **a** The number of putative precursors of class II bacteriocins detected by hmmsearch and BAGEL4. They identified 128,369 and 90,026 putative precursors, respectively, with 30,746 sequences in common. **b** Distribution of the length of 2,005 representative precursors, which cd-hit designated. **c** Rarefaction curve of clusters of class II bacteriocin precursors (blue line). The Red dashed line shows that 1,775 sequences belonging to 188 clusters were highly similar to known class II bacteriocins (identity > 90%, coverage > 95%). **d** Number of clusters in different genera (left), species (middle), and genomes (right). **e**, t-SNE plot reveals the distinct profile of class II bacteriocins in different body sites. Each dot represents one metagenome sample. **f** The prevalence and average abundance of 644 precursor clusters detected in six body sites. Each dot denotes one precursor cluster. The abundance in the individual is shown in Supplementary Fig. 17. The red numbers are the number of precursor clusters with a log2-transformed average abundance > -5 (shown in red dashed line) and the number of precursor clusters detected in each body site

The findings of the antagonistic potential of class II bacteriocins and their variable prevalence and predominance in the human microbiome raise the question of what extent the class II bacteriocins may link to microbiome homeostasis. We next attempted to group class II bacteriocins into subfamily with similar biological functions based on precursor sequence space and investigate their profiling in the human microbiome in detail*.* To fully reveal the chemical diversity of class II bacteriocin, we first adapted two approaches for the identification of precursor peptides, with hmmsearch [35] to search Pfam domains of precursors of class II bacteriocin (Supplementary Table 4) and with BAGEL4 [36] which is a tool specifically designed for bacteriocin mining. We combined two approaches to identify 187,649 precursors from class II bacteriocin BGCs (Fig. 5a, Supplementary Table 8). We then grouped 187,649 putative precursors into 2,005 clusters with a threshold of 50% sequence identity (Supplementary Fig. 16). The sequence lengths of those representative precursors were approximately normally distributed, with a center of ~ 55 amino acids (Fig. 5b). The accumulation curve showed that the precursor diversity increased with the number of genomes included, indicating more class II bacteriocins will be disclosed with more genomes sequenced (Fig. 5c). Moreover, we found that only 188 clusters were similar to 333 known class II bacteriocins (identity > 90%, coverage > 95%), leaving the vast majority (1,817/2,005) underexplored (Supplementary Table 9). Of note, while the rule-based method hmmsearch and BAGEL4 enable a high likelihood of positive detection, at the same time, they probably underestimate the real biosynthetic potentials of bacteriocins.

In line with the taxa-specificity of the GCFs, most class II bacteriocin precursors family were genus-specific (*n* = 1,862, 92.9%) and species-specific (*n* = 1,327, 66.2%), with 33.7% of precursor family being even strain-specific (*n* = 675) (Fig. 5d). We next examined their profile in the human microbiome. Of 644 clusters detected in six body sites, about 31.8% of clusters were niche-specific in one of six sites (Supplementary Fig. 17a). Moreover, the profiles of class II bacteriocins in different body sites were distinct as revealed by t-SNE plot, further supporting the niche-specificity of class II bacteriocin in the human microbiome (Fig. 5e). Their profiles in vagina and skin showed great individual variations, whereas the class II bacteriocins in other body sites were relatively conserved with being clustered together. Additionally, those class II bacteriocins were sporadically present in the skin and gut, whereas some class II bacteriocins were particularly enriched in the oral cavity and vagina with a high prevalence and abundance (Fig. 5f, Supplementary Fig. 17b). Probably due to the individual variations in the vagina, a member of subfamilies of class II bacteriocins exhibited a relatively smaller prevalence in the vagina than in the oral cavity. Both the GCFs profile (Fig. 3d) and precursors profile (Figs. 5e, f) in the human microbiome suggested that class II bacteriocins are particularly enriched in the vaginal microbiome. Considering the vagina has simple communities with the lowest alpha diversity than other body sites [37], we reasoned that those enriched and predominant class II bacteriocins might play prominent roles in regulating microbial community in the vagina.

### Multi-omics analysis reveales class II bacteriocins potentially contributing to vaginal microbiome homeostasis

**Fig. 6:**
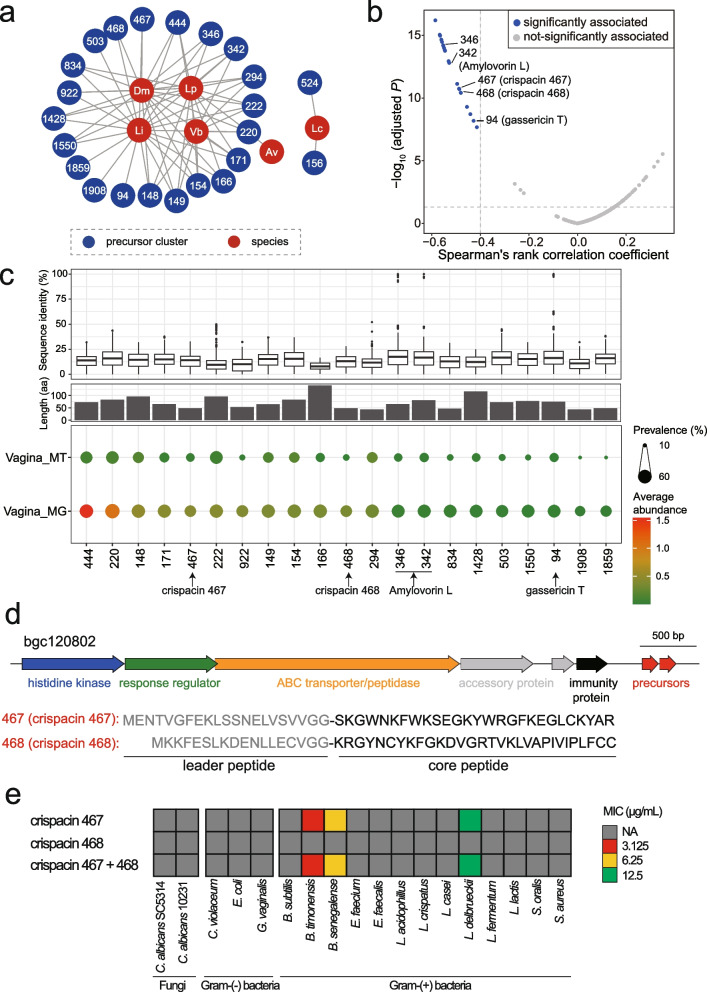
Antagonistic class II bacteriocins potentially play a regulatory role in the vaginal microbiome. **a** Correlation network between precursor clusters of class II bacteriocins and bacterial species in the vaginal microbiome. 23 clusters are negatively correlated with species in the community, with spearman’s *rho* < -0.3 and adjusted* P* < 0.05 shown in the network. The number in the node denotes the precursor cluster number. Av, *Atopobium vaginae*; Dm, *Dialister micraerophilus*; Lc, *Lactobacillus crispatus*; Li, *Lactobacillus iners*; Lp, *Lactobacillus paragasseri*; Vb, *Veillonellaceae bacterium* DNF00626. **b** Spearman correlation between precursor clusters and alpha diversity (Shannon index). The dashed line denotes the correlation coefficient cutoff < -0.4 and adjusted *P* < 0.05. Points refer to 240 precursor clusters detected in the vaginal metagenomes, 21 of which were significantly associated with the alpha diversity of the vaginal microbiome. **c** Global sequence identity to known class II bacteriocins (upper), sequence length (middle), abundance and prevalence of 21 clusters (bottom) in the vaginal metagenome (MG, *n* = 169) and metatranscriptome (MT, *n* = 180). The point size and color are proportionate to the cluster prevalence and average abundance in the MG and MT datasets, respectively. **d** Gene organization of bgc120802 and precursor sequences of cluster_467 and cluster_468. Putative double-glycines leader peptides are in grey. **e** The minimum inhibitory concentration (MIC) of chemically synthesized bacteriocins. NA: not available, no inhibitory effect detected with 200 μg/mL. Gram-(-) bacteria, Gram-negative bacteria; Gram-( +) bacteria, Gram-positive bacteria

To examine the class II bacteriocins that may account for the homeostasis of the vaginal microbiome, we first constructed the association network between class II bacteriocins and bacterial species at the metagenomic level. We found 23 precursor clusters correlated negatively with various species, indicating their antagonistic potential in regulating the vaginal microbiome (Fig. 6a). In particular, 21 clusters were negatively correlated with *Lactobacillus iners*, which is more conducive to the occurrence of abnormal vaginal microflora and thus a potential new therapeutic target for bacterial vaginosis treatment. Additionally, 21 of 23 clusters were also found to be inversely correlated to the Shannon index (Spearman *rho* < -0.4, adjusted *P* < 0.05) (Fig. 6b). Lower bacteria diversity in the microbiome with these detected class II bacteriocins suggested their regulative role in shaping the microbiome. To confirm whether these antagonistic bacteriocins are biologically functional, we next inspected their expression profile in the 180 metatranscriptomic datasets (Supplementary Table 6) and found that most of them were actively transcribed in the vaginal microbiome of healthy individuals (Fig. 6c). Three of the 21 clusters grouped with known class II bacteriocins, including Amylovorin L (cluster_342 and cluster_346, a two-component class IIb bacteriocin) from *Lactobacillus amylovorus* DCE 471 [38] and gassericin T (cluster_94) from *Lactobacillus gasseri* SBT2055 [39]. The findings of known class II bacteriocins with protective roles by omics-based association analysis further validated the effectiveness of our approach in discovering the regulatory bacteriocins in the microbiome. The other 18 clusters of class II bacteriocins were also prevalent and actively transcribed in the vaginal microbiome but uncharacterized yet.

We next sought to validate the antagonistic potential of those uncharacterized bacteriocins experimentally. For a proof of principle, we selected two precursor clusters (cluster_467 and cluster_468) with high abundance and a short peptide length that make their chemical synthesis practical. Those two precursors were located on BGCs (e.g., bgc120802) identified from 16 genomes of *L. crispatus* (Supplementary Fig. 18). The bgc120802 harbors specific class II bacteriocins-related genes, including a two-component regulator system (histidine kinase and response regulator), ABC transporter, and immunity protein. The precursor sequences from 16 BGCs were identical and featured a canonical double-glycine leader (Fig. 6d, Supplementary Fig. 18). We thus synthesized the core peptides (Supplementary Fig. 19), namely crispacin 467 (27 amino acids) and crispacin 468 (30 amino acids), respectively, and validated their antagonistic activity toward bacteria and fungi. The antimicrobial assay showed that the crispacin 467 exhibited antibacterial activities against three Gram-positive bacterial strains (i.e., *Bacillus timonensis*, *Brevibacterium senegalense*, and *L. delbrueckii* subsp. *bulgaricus*) with a minimum inhibitory concentration of 3.125, 6.25 and 12.5 μg/mL, respectively. However, the inhibitory effects of crispacin 468 were not observed, nor a synergy of them (Fig. 6e). The crispacin 468 might exhibit antimicrobial activity against other species beyond the tested strains. Taken together, we believe that the bacteriocin producers arm the vagina microbiome with diverse antagonistic bacteriocins, potentially preventing pathogen invasion and stabilizing the microbial community. Though how LAB employs SMs to shape their microbiome communities is not yet fully understood, our omics-guided discovery of new bacteriocins from LAB provides an alternative way to the discovery of new antibacterial therapeutics for microbiome dysbiosis.

## Discussion

Although the health-promoting effects of LAB in the human microbiome are increasingly recognized, their interplay with other microbes and their influence on microbiome homeostasis are still not well understood. Previous biosynthetic analysis of LAB in a limited dataset focuses on particular metabolites, proposing their protective roles to host [40, 41]. However, the landscape of LAB SMs, particularly their profiles and potential roles in the human microbiome, remains elusive. In this study, we conducted a comprehensive omic analysis for LAB BGCs, significantly enhancing our understanding of the diversity and distribution of LAB BGCs. We found 129,878 BGCs of 2,849 GCFs from 31,977 LAB genomes, most of which were species-specific and encoded diverse uncharacterized SMs. We further investigated human metagenomes of six body sites to disclose the BGCs profile of LAB in the human microbiome, revealing that the diverse LAB SMs are variably prevalent in the human microbiome. Of note, BGCs of class II bacteriocins were particularly enriched and predominant in the vaginal microbiome. The niche specificity of GCFs in the human microbiome together with their species- or even strain-specificity, suggested that the LAB SMs may provide a competitive advantage to the niche adaptation of their producing hosts.

In this study, we employed a well-established method of grouping BGCs into families and clans based on architectural relationships to profile BGCs in the human microbiome, allowing us to prioritize novel BGCs for natural product discovery [24, 42]. Although informative, the GCF grouping will be affected by the imperfect BGC boundary prediction of antiSMASH [21]. Most LAB SMs were predicted to be antibacterial using machine learning models, indicating their potential regulatory roles in the human microbiome and putative protective roles for the host. However, due to limited training data, our machine learning model can only predict limited bioactivities (antibacterial, antifungal, and antitumor), underestimating other biological potentials of LAB SMs. Although such evidence does not exclude the possibility of other biological functions, we believe that antagonistic LAB SMs, particularly bacteriocins, potentially provide competitive edges to their producers and regulate the microbiome community.

Applying metagenomics and metatranscriptomics analysis, we underscored 21 class II bacteriocins actively expressed in the vaginal microbiome and negatively correlated with individual bacteria species. Together with their negative association with the α-diversity of the vaginal microbiome, we can envision that these bacteriocins play prominent roles in regulating homeostasis. As proof of principle, we identified a novel class II bacteriocin produced by *L. crispatus*, namely crispacin 467, which exhibited potent antibacterial activities against Gram-positive bacterial strains. While previous analyses have disclosed the bacteriocin biosynthetic genes and antibacterial activity in *L. crispatus* [43, 44], little is known regarding their antagonistic bacteriocin except for crispacin A [45]. Furthermore, the potential roles of bacteriocins in shaping the microbiome remain largely unexplored. The human microbiome harbors a vast array of class II bacteriocins in *Lactobacillus* and other LAB, providing significant potential for discovering new antimicrobials. While the precursors of 21 bacteriocins were prevalent and transcribed in the vaginal microbiome, whether they are produced in situ in the vagina still needs to be examined by metabolomics. Moreover, how LAB employ SMs to shape their microbiome communities requires further exploration using in vivo mouse models or in vitro polymicrobial models. Nonetheless, the findings from our study suggest that LAB can utilize bacteriocins to regulate bacterial interactions, which plays a critical role in maintaining microbial community composition and promoting microbiome homeostasis. It is important to note that the health benefits of *Lactobacillus spp.* are not solely attributed to the pH-lowering effect of lactic acid. While the acidification of the vagina can inhibit the growth of pathogenic and non-*Lactobacillus* microbes (such as *Gardnerella*, *Prevotella*, and *Mobiluncus*), other factors, such as the production of bacteriocins and hydrogen peroxide (H_2_O_2_) also contribute to promoting vaginal health [46, 47]. It is plausible that *L. crispatus*-produced bacteriocins, along with the low pH and H_2_O_2_ production, fine-tuned the vaginal microbial community and promoted a healthy vaginal ecosystem [47].

LAB, especially the genus *Lactobacillus*, dominate the existing probiotics that confer a health benefit on the host when administered adequately [48]. The beneficial effects of probiotics result from diverse mechanisms, among which is bacteriocin production. Antagonistic bacteriocins that assist the producer colonization and provide protective roles for the host are important for probiotics to offer beneficial effects. Our findings reinforced the understanding of pervasive bacteriocins of LAB, which are also widespread in the human microbiome, particularly in the vagina. Those LAB and their bacteriocins present in healthy individuals are promising priorities for microbiome-based therapeutics [49]. For example, with the potential to modulate the vaginal microbiota, probiotics containing *Lactobacillus* spp. have been applied to treat bacterial vaginosis [47]. Moreover, the diverse antagonistic bacteriocins could be borrowed and arm genetically engineered beneficial LAB probiotics with a therapeutic property [50], preventing the host from the pathogen invasion directly or indirectly (via microbiota- and/or immune modulation). Continued investigation of the biosynthetic capacity and ecological roles will help to facilitate the translation of LAB and their antagonistic SMs into clinical application.

In summary, our study provides a global insight into the biosynthetic potentials of LAB SMs and a starting point for the omics-guided discovery of antagonistic SMs that potentially regulate microbiome homeostasis. Class II bacteriocin predominant in vaginal microbiome but negatively associated with its bacterial diversity is experimentally validated to play antagonistic roles in microbial communities. To the best of our knowledge, our study is the first to systematically unveil LAB SM biosynthetic potentials and their profile in the healthy human microbiome. However, the analysis presented here cannot be considered exhaustive. The machine learning strategies employed to predict the bioactivity of SMs remain refined by knowledge accumulation of LAB SMs and their biosynthesis and bioactivity. Additionally, how LAB employ SMs to shape their microbiome communities in the human niche remains to be studied. Nevertheless, our systematic investigation of the biosynthetic potential of LAB provides a good starting point for the omics-guided discovery of SMs with therapeutic potential from the human microbiome. In addition to enhancing our understanding of the profile of LAB SMs and their potential regulating roles in the human microbiome, the discovery of antagonistic bacteriocins opens up exciting opportunities for future research on various probiotic applications of LABs.

## Methods

### Data acquisition

As defined early, lactic acid bacteria include 14 genera, comprising *Lactobacillus*, *Lactococcus*, *Leuconostoc*, *Pediococcus*, *Streptococcus*, *Aerococcus*, *Alloiococcus*, *Carnobacterium*, *Dolosigranulum*, *Enterococcus*, *Oenococcus*, *Tetragenococcus*, *Vagococcus*, and *Weissella* [51]. Particularly, the genus *Lactobacillus* has been reclassified recently [52], extending to 25 genera consisting of *Lactobacillus*, *Paralactobacillus, Amylolactobacillus, Acetilactobacillus*, *Agrilactobacillus*, *Apilactobacillus*, *Bombilactobacillus*, *Companilactobacillus*, *Dellaglioa*, *Fructilactobacillus*, *Furfurilactobacillus*, *Holzapfelia*, *Lacticaseibacillus*, *Lactiplantibacillus*, *Lapidilactobacillus*, *Latilactobacillus*, *Lentilactobacillus*, *Levilactobacillus*, *Ligilactobacillus*, *Limosilactobacillus*, *Liquorilactobacillus*, *Loigolactobacilus, Paucilactobacillus*, *Schleiferilactobacillus*, and *Secundilactobacillus*. Filtered with taxonomy, genomes from these 38 genera were then retrieved from the NCBI reference sequences (RefSeq) database [16] (as of Aug. 2021, including SAGs only), PATRIC database (including SAGs) [17], IMG/M database (including SAGs and MAGs) [18]. Besides them, genomes from two previous studies focusing on the human gut microbiome (including SAGs and MAGs) [19] and food-originated LAB (including MAGs) [20] were also included. To avoid reference genome redundancy, genomes from RefSeq were compared to themselves and those from other sources using Mash v2.3 [53]. Genomes with a Mash distance of 0 were considered identical. Only the one with a minimal number of contigs was retained. As potential misclassification might be present, GTDB-Tk v1.7.0 [54] was further used to confirm and unify taxonomic annotation against GTDB-Tk reference data version r202 [55]. There is a slight difference between the NCBI taxonomy and GTDB taxonomy [56]. Under GTDB taxonomy, five genera are subdivided: *Carnobacterium*, *Enterococcus*, *Lactococcus*, *Vagococcus*, and *Weissella*. Finally, a total of 56 genera belonging to six families (Lactobacillaceae, Aerococcaceae, Streptococcaceae, Vagococcaceae, Enterococcaceae, and Carnobacteriaceae) were considered members of LAB in this study.

### Biosynthetic gene cluster analysis

Biosynthetic gene clusters for each genome were annotated by antiSMASH 6.0 [21] with default parameters. In total, 31,977 LAB genomes and 164,417 non-LAB genomes (the intersection between RefSeq and GTDB repository version r202) [56] were included for BGC annotation. This resulted in 130,051 BGCs from 30,718 LAB genomes and 1,122,204 BGCs from 155,540 non-LAB genomes. No BGCs were annotated in 1,259 LAB genomes and 8,877 non-LAB genomes.

### Clustering BGCs into families and clans

BiG-SLiCE [22], a tool to cluster sizable BGCs, contains two BGC features (biosynthetic-Pfam and sub-Pfam domains). Those BGC features are sufficient to distinguish distinct BGC classes. As a previous study described [25], all features of LAB BGCs and 1910 experimentally validated BGCs from the MIBiG 2.0 repository were extracted by BiG-SLiCE v1.1.0 and subsequently used to compute all-to-all cosine distances between BGCs using Python suite SciPy version 1.6.2 [57]. The cosine distances were next subjected to hierarchical clustering with average linkage, grouping BGCs into families (GCFs, distances < 0.2) and clans (GCCs, distances < 0.8) by Python 3.8 with Scikit-learn version 0.24.2 [58].

### Metagenomics and metatranscriptomics analysis

The raw metagenomic sequencing reads of 748 HMP samples [30] and the raw metatranscriptomic data of 180 vaginal samples [59] were acquired from NCBI SRA (Sequence Read Archive) [60] under project accession number PRJNA48479 and PRJNA797778, respectively. Fastp 0.21.1 [61] with default parameters was adopted for detecting and removing low-quality sequencing reads. High-quality metagenomic sequencing reads were subjected to kneaddata (https://github.com/biobakery/kneaddata) for discarding reads belonging to the human host, through searching against the human reference genome (GRCh38.p13) from GENCODE [62]; high-quality metatranscriptomic reads were also subjected to SortMeRNA v4.3.4 [63] for removing reads derived from ribosomal RNAs. Following that, MetaPhlAn v3.0.13 [64] was used for taxonomic profiling. Prior to assessing the abundance of BGCs in metagenomics and metatranscriptomic data, we used a modified script from BiG-MAP [65] to de-duplicate 130,051 BGCs. To reduce the computational load, we de-duplicated them within each GCF, at a 0.8 nucleotide identity threshold, leading to 24,222 non-redundant BGCs, the nucleotide sequences of which were used to generate the reference database. Next, the non-host metagenomic and metatranscriptomic reads were mapped to this BGC reference using Bowtie 2 v2.3.5.1 [66], with a parameter of “-k 1”. We then utilized featureCounts v2.0.3 [67] (with parameters of “-T 30 -f -p -B -C -t CDS -g ID -M -O –fracOverlap 0.2”) to assign sequencing reads to the BGC genes. When calculating the abundance of a BGC, we only considered the core and additional biosynthetic genes, excluding the other genes such as transporters, regulators, transposases, and so forth. For each BGC, a corresponding GTF (General Transfer Format) annotation file was generated by antiSMASH. We retrieved the biosynthetic-related genes (tag “biosynthetic” for the core biosynthetic genes and “biosynthetic-additional” for the additional biosynthetic genes) according to the “gene_kind” tag in GTF files. A BGC was considered present in a metagenomic sample when fulfilling the following criteria: (1) the percentage of biosynthetic-related genes detected is over 50% of total biosynthetic-related genes in a BGC; (2) at least one core biosynthetic gene was found in a BGC. The abundance of a BGC was computed via the Eq. (1):

1 $$\mathrm{BGC\ abundance}=\frac{{\sum }_{i=1}^{k}\frac{{N}_{i}}{{L}_{i}}}{k \times N} \times {10}^{6}$$

N_i_ represents the number of reads mapped on a biosynthetic-related gene; L_i_ represents the gene length; k represents the number of biosynthetic-related genes in a BGC; N represents the total number of high-quality non-host reads in a metagenome/metatranscriptome sample.

### Prediction of secondary metabolite activity

To predict the activity of BGC-encoding compounds, we used mlr v2.19.0 [68] to perform machine learning. The training dataset comprising 950 MIBiG BGCs with known activities (antibacterial, antifungal, antitumor or cytotoxic, or other activities) was gathered by Walker et al*.* [32]*.* BGC features of those known BGCs were extracted by BiG-SLiCE [22]. Prior to training models, we removed BGC features present in < 10 BGCs. Rather than multiclass classification, binary classification was adopted for each activity class since a molecule might have multiple functions. Four two-class classifiers (namely, logistic regression, elastic net regression, random forest, and support vector machines) were adopted for binary classification of the activities of BGC products. In order to obtain the honest performance of four classifiers, we performed a nested resampling for parameter tuning with threefold cross-validation in the inner and tenfold cross-validation in the outer loop, in which a random search with a maximum iteration of 50 was adopted. This would generate 30 instances for each classifier. The average AUROC was used to evaluate the performance of four classifiers. The function *generateThreshVsPerfData* is used to calculate one or several performance measures, such as the false positive rate (fpr) and true positive rate (tpr), for a range of decision thresholds from 0 to 1. The resulting fpr and tpr values for each threshold are plotted on a receiver operating characteristic (ROC) curve, which allows us to estimate the performance of a classifier. Using the random forest model, 129,878 LAB-derived BGCs and 1,121,156 non-LAB-derived BGCs containing BGC features were subjected to activity prediction. Chord diagram showing the association between BGC classes and predicted activities of their products was plotted using R package *circlize* v0.4.13 [69]. Sankey diagram showing the association between species and BGC classes was done by package *networkD3* v0.4 [70].

### Precursor of class II bacteriocins

In order to pinpoint the precursors of class II bacteriocins, we first used Prodigal-short [71] to identify all small ORFs. We then used hmmsearch [35] to search class II bacteriocins-related domains (provided in Supplementary Table 4) against ORFs of all RiPP-like BGCs. The hits with a threshold of *E*-value < 0.01 were considered as the precursors of class II bacteriocins. Meanwhile, BAGEL4 [36] was also adopted for searching class II bacteriocins from RiPP-like BGCs. They detected 128,599 and 90,101 putative precursors, respectively, with 30,764 in common. We discarded 287 sequences that were larger than 150 amino acids (AAs), retaining 187,649 sequences for further analysis. Those sequences were then grouped into clusters using Cd-hit [72], with the parameters of “-n 2 -p 1 -c 0.5 -d 200 -M 50,000 -l 5 -s 0.95 –aL 0.95 –g 1”. The sequences with an identity of > 50% will be grouped into one cluster, as proteins with > 50% identity generally share a common function [73]. To collect the known class II bacteriocins, we queried NCBI PubMed with the keyword “class II bacteriocin”. Meanwhile, we also included the sequences gathered by Yi et al*.* [15] as well as the sequence deposited in the BAGEL4 database [36]. In total, 333 sequences of class II bacteriocins were obtained (Supplementary Table 9). As the curated 333 sequences might be the mature peptides, a local sequence aligner, DIAMOND v2.0.15 [74], was utilized to compare 333 known class II bacteriocins to 187,649 precursor sequences with the parameter of “–id 90 –query-cover 95 –masking 0”. The known class II bacteriocins showed an alignment of identity > 90% and coverage > 95% with 1,775 precursor sequences belonging to 188 clusters that were thus regarded as homologous. For 21 selected precursor clusters, we identified the global identity relative to the known class II bacteriocins using the Needleman-Wunsch algorithm in the function “needleall” of EMBOSS software package [75]. The alignment of precursors was done by MAFFT v7.490 [76] with the parameter of “–maxiterate 1000 –localpair”, and then was visualized using Jalview software [77]. To conveniently inspect the gene organizations of BGCs harboring precursors of cluster_467 and cluster_468, we adopted BiG-SCAPE [24] for exploring their architectures.

Precursor abundance in metagenome/metatranscriptome samples was computed via the Eq. (2):

2 $$\mathrm{precursor\ abundance}=\frac{{N}_{i}}{{L}_{i} \times N} \times {10}^{6}$$

Here, N_i_ represents the number of reads mapped on a precursor gene; L_i_ represents the gene length; N represents the total number of high-quality non-host reads in a metagenome/metatranscriptome sample. The abundance of a precursor cluster is the sum of the abundance of precursors in this cluster.

### Phylogenetic tree construction

GTDB repository version r202 [56] contains 822 representative genomes of 56 LAB genera. The genome with the largest N50 length in each genus was selected as a proxy for its corresponding genus. Consistent with the previous approach to constructing bacterial reference trees [56], the multiple sequence alignment of the concatenation of 120 phylogenetically informative marker genes of 56 representative genomes was used to infer the phylogenetic tree. IQ-TREE version 2.1.4-beta [78] was adapted for constructing maximum likelihood (ML) phylogenetic trees, with 1,000 ultrafast bootstrap replicates. In-built ModelFinder [79] identified the best-fit model as LG + F + R8. Inferred phylogeny was visualized using iTOL [80].

### Peptide synthesis

The two deducted core peptides of cluster_467 and cluster_468 were chemically synthesized by Sangon Biotech (Shanghai, China). Their molecular weights were confirmed by mass spectrometry, and their required purity was ≥ 90%, determined by high-performance liquid chromatography. The synthesized peptide powder was stored at − 80 °C and dissolved in sterilized double-distilled water to 5 mg/mL upon use.

### Bacterial and fungal strains

A total of 29 bacterial strains and two fungal strains were used in this study (Supplementary Table 10). Their growth conditions are as follows: two bacterial strains (*Escherichia coli* DH5α, *Staphylococcus aureus* B04) were incubated in Luria–Bertani (LB) culture medium at 37 ℃ under 180 rpm rotation; 13 bacterial strains (*Chromobacterium violaceum*, *Bacillus subtilis* 168, *Enterococcus faecium* MCC2763, *Enterococcus faecalis* OG1RF, *Enterococcus caccae* DSM 19114, *Enterococcus ureasiticus* DSM 23328, *Enterococcus haemoperoxidus* DSM 15920, *Enterococcus silesiacus* DSM 22801, *Enterococcus termitis* DSM 22803, *Enterococcus wangshanyuanii* DSM 104047, *Enterococcus rivorum* DSM 104544, *Brevibacterium senegalense* DSM 25783, *Rothia sp.* Olga DSM 111809) were incubated in tryptic soy broth (TSB) medium at 30 ℃ with shaking at 180 rpm; *Bacillus timonensis* DSM 25372 was grown in trypticase soy agar (TSA) at 30 ℃; *Lactococcus lactis* subsp. *cremoris* MG1363 was grown statically at 30 ℃ in M17 medium; 11 bacterial strains (including *Streptococcus oralis* subsp. *tigurinus*, *Lactobacillus delbrueckii* subsp. *bulgaricus*, *Lactobacillus crispatus* ATCC 33820, *Lactobacillus acidophillus* ATCC 9224, *Lactobacillus casei* ATCC 393, *Lactobacillus fermentum* ATCC 14932, *Latilactobacillus sakei* subsp. *sakei* DSM 6333, *Lactiplantibacillus plantarum* DSM 20174, *Lactobacillus gasseri* DSM 20243, *Limosilactobacillus reuteri* DSM 20016, *Leuconostoc mesenteroides* subsp. *dextranicum* DSM 20484) were incubated statically in deMan, Rogosa and Sharpe (MRS) medium at 30 ℃ or 37 ℃; *Gardnerella vaginalis* ATCC 14018 was maintained in Colombia blood agar at 37 ℃ in anaerobic conditions, and the suspension culture was grown by taking a loop full of colonies from the agar plate and incubating in Brain heart infusion broth (BHI), at 37 ℃ in anaerobic conditions; two fungal strains (*Candida albicans* SC5314, *Candida albicans* ATCC 10231) were grown in Roswell Park Memorial Institute (RPMI) media at 37 ℃ with shaking at 150 rpm.

### Agar well diffusion assay

Indicator strains were cultivated overnight. Around 40 μL microbial inoculum was blended with the 20 mL corresponding agar medium before solidifying. Following solidifying, a hole with a diameter of around 6 mm was punched with a sterile tip, and a 10 µL of crispacin 467 and/or 468 to a final amount of 0.05 mg was introduced into the well. All agar plates were incubated at their corresponding growth conditions for 1–2 days.

### Determination of minimum inhibitory concentrations

The minimum inhibitory concentrations (MICs) of the two peptides [individually and in combination (1:1 ratio)] against bacterial and fungal strains were performed by broth microdilution. Tested bacterial strains were inoculated overnight in the corresponding culture medium (LB, M17, TSB, TSA, BHI, or MRS) and at respective growth conditions. The optical density at 600 nm (OD_600_) of bacterial cultures was determined to estimate the bacterial concentration. The bacteria cultures were diluted to ~ 5 × 10^5^ CFU/mL using the respective broth. 100 μL aliquots of bacterial suspensions were transferred into 96-well plates containing two-fold serial dilutions of peptides (ranging from 200 μg/mL to 0.19 μg/mL). After incubating for 24 h, bacterial growth was assessed by determining OD_600_. Besides, MIC against the fungal strains was determined according to the CLSI M27-A3 guidelines [81]. Briefly, *C. albicans* strains were cultured overnight in RPMI medium and grown fungal suspensions were centrifuged at 5,000 rpm for 10 min and the pellet was resuspended and washed twice with 1 × PBS to remove the dead cells. The fungal inoculum was standardized to 1 × 10^6^ CFU/mL using a spectrophotometer and added to the well plate containing varying concentrations of the peptide (200 μg/mL to 0.19 μg/mL). The media without the peptide served as a control. The plates were then incubated at 37 °C for 24 h with shaking at 80 rpm, and the absorbance was measured at 520 nm using SpectraMax 340 tunable microplate reader (Molecular Devices, San Jose, CA, USA). The MIC value was determined as the lowest concentration of the peptides where no bacterial or fungal growth was detected. All assays were conducted in triplicate on three independent occasions.

### Statistical analysis and visualization

The accumulations of GCFs detected in metagenomes as well as clusters of class II bacteriocin precursors were computed with function *specaccum* in R package *vegan* v2.5–7 [82]. R package *UpSetR* v1.4.0 [83] was adopted for visualizing the intersection of GCFs or precursor clusters detected in different body sites. Chi-squared test and wilcoxon rank-sum test (two sided) were done by function *chisq.test* and *wilcox.test* in R, respectively. The alpha diversity (Shannon index) of the vaginal microbiome was calculated with R package *vegan* v2.5–7 [82]. To visualize the distribution of class II bacteriocins detected in six body sites, a dimensionality reduction was performed using t-distributed stochastic neighbor embedding (t-SNE), which was done by R package *Rtsne* v0.15 [84]. Spearman's correlations between precursor clusters vs. bacterial species and between clusters vs. Shannon index were computed with the function *corr.test* in R package *psych* v2.1.9 [85], and *P* values were adjusted with the “BH” method [86]. The heat maps in this study were plotted using package *pheatmap* v1.0.12 [87]. Cytoscape 3.9.0 [88] was used to visualize the network of similarity of class II bacteriocins and the network of species-precursor correlation. Without a specific statement, other figures were generated using ggplot2 v3.3.5 [89]. All statistical analyses were finished in R v4.1.2.

## Supplementary Information

**Additional file 1: Supplementary Table 1.** LAB genomes collected in this study. **Supplementary Table 2.** List of 31977 LAB genomes. **Supplementary Table 3.** List of 130051 LAB BGCs. **Supplementary Table 4.** Information of bacteriocins-related domains. **Supplementary Table 5.** List of 3805 non-LAB genera. **Supplementary Table 6.** List of metagenomic and metatranscriptomic data used in this study.** Supplementary Table 7.** Information of 950 known BGCs adopted as train dataset. **Supplementary Table 8.** Putative precursors of class II bacteriocins. **Supplementary Table 9.** List of 333 known class II bacteriocins. **Supplementary Table 10.** Inhibition spectrum of crispacins against 31 indicator strains in agar well diffusion assay.

**Additional file 2: Supplementary Figure 1. **Overview of the data processing.** Supplementary Figure 2. **Number of BGCs identified from MAGs and SAGs.** Supplementary Figure 3. **The number of bacteriocins identified from 72,471 RiPP-like BGCs.** Supplementary Figure 4. **Biosynthetic potential in LAB species.** Supplementary Figure 5. **Median BGC count and proportion in SAGs.** Supplementary Figure 6. **Comparison of BGC proportion and counts in LAB.** Supplementary Figure 7. **Comparison of SM BGC capacity between LAB and non-LAB genera.** Supplementary Figure 8. **Distribution of 2,849 GCFs.** Supplementary Figure 9. **Diversity of 212 cross-genus GCFs.** Supplementary Figure 10. **Domain distribution of 88 cross-genus RiPP-like GCFs.** Supplementary Figure 11. **BGCs are prevalent in six body sites.** Supplementary Figure 12. **Distribution of reference BGCs with different activities in training data.** Supplementary Figure 13. **Performances of four classifiers in determining activities of BGC-encoding compounds.** Supplementary Figure 14. **Profile of predicted compound activities of BGCs in LAB genera.** Supplementary Figure 15. **The predicted activity of cytotoxic and antifungal.** Supplementary Figure 16. **The sequence similarity network of precursor peptides reveals the huge diversity of putative class II bacteriocins.** Supplementary Figure 17. **The variable prevalence of class II bacteriocins in six body sites.** Supplementary Figure 18. **BGCs harboring precursors of cluster_467 and cluster_468.** Supplementary Figure 19. **HR-LCMS analysis of synthesized peptides.

## Data Availability

The bacterial genomes are publicly available in NCBI Assembly RefSeq database (https://ftp.ncbi.nlm.nih.gov/genomes/refseq/), PATRIC database (https://docs.patricbrc.org/user_guides/ftp.html), and IMG/M database (https://img.jgi.doe.gov/cgi-bin/m/main.cgi). The genomes from human gut are available in the European Nucleotide Archive under study accession ERP116715, and genomes from food metagenomes are available at http://www.tfm.unina.it/DATA001-2020-Pasolli. Genomes can be obtained through the accession numbers provided in Supplementary Tables 1 and 5. The raw data for HMP metagenomes [30] and vaginal metatranscriptomes [59] are publically available in NCBI-SRA under the BioProjects PRJNA48479 (https://www.ncbi.nlm.nih.gov/bioproject/48479) and PRJNA797778 (https://www.ncbi.nlm.nih.gov/bioproject/?term=PRJNA797778), respectively. The samples used in this study are provided in Supplementary Table 6. The analysis codes supporting this study’s findings are available on the GitHub repository (https://github.com/ZhangDengwei/LAB_BGCs).
