# Supplementary material for: A systematically biosynthetic investigation of lactic acid bacteria reveals diverse antagonistic bacteriocins that potentially shape the human microbiome
Source: Microbiome. 2023 Apr 27;11:91. doi: 10.1186/s40168-023-01540-y (PMC10134562; doi:10.1186/s40168-023-01540-y)
Supplement: Supplementary file 3 — Additional file 2: Supplementary Figure 1. Overview of the data processing. Supplementary Figure 2. Number of BGCs identified from MAGs and SAGs. Supplementary Figure 3. The number of bacteriocins identified from 72,471 RiPP-like BGCs. Supplementary Figure 4. Biosynthetic potential in LAB species. Supplementary Figure 5. Median BGC count and proportion in SAGs. Supplementary Figure 6. Comparison of BGC proportion and counts in LAB. Supplementary Figure 7. Comparison of SM BGC capacity between LAB and non-LAB genera. Supplementary Figure 8. Distribution of 2,849 GCFs. Supplementary Figure 9. Diversity of 212 cross-genus GCFs. Supplementary Figure 10. Domain distribution of 88 cross-genus RiPP-like GCFs. Supplementary Figure 11. BGCs are prevalent in six body sites. Supplementary Figure 12. Distribution of reference BGCs with different activities in training data. Supplementary Figure 13. Performances of four classifiers in determining activities of BGC-encoding compounds. Supplementary Figure 14. Profile of predicted compound activities of BGCs in LAB genera. Supplementary Figure 15. The predicted activity of cytotoxic and antifungal. Supplementary Figure 16. The sequence similarity network of precursor peptides reveals the huge diversity of putative class II bacteriocins. Supplementary Figure 17. The variable prevalence of class II bacteriocins in six body sites. Supplementary Figure 18. BGCs harboring precursors of cluster_467 and cluster_468. Supplementary Figure 19. HR-LCMS analysis of synthesized peptides. [file 40168_2023_1540_MOESM2_ESM.docx]

**A systematically biosynthetic investigation of lactic acid bacteria reveals diverse antagonistic bacteriocins that potentially shape the human microbiome**

Dengwei Zhang^1^, Jian Zhang^1^, Shanthini Kalimuthu^2^, Jing Liu^1^, Zhiman Song^1^, Bei-bei He^1^, Peiyan Cai^1^, Zheng Zhong^1^, Chenchen Feng^3^, Prasanna Neelakantan^2^, Yong-Xin Li^1, *^

^1^ Department of Chemistry and The Swire Institute of Marine Science, The University of Hong Kong, Pokfulam Road, Hong Kong, China

^2^ Division of Restorative Dental Sciences, Faculty of Dentistry, The University of Hong Kong, Hong Kong, China

^3^ Department of Urology, Huashan Hospital, Fudan University, Shanghai 200040

^*^To whom correspondence may be addressed.

Email: [yxpli@hku.hk](mailto:yxpli@hku.hk)

**Supplementary Figures:**

[Supplementary Figure 1. Overview of the data processing. 3](#_Toc107933259)

[Supplementary Figure 2. Number of BGCs identified from MAGs and SAGs. 4](#_Toc107933260)

[Supplementary Figure 3. The number of bacteriocins identified from 72,471 RiPP-like BGCs. 5](#_Toc107933261)

[Supplementary Figure 4. Biosynthetic potential in LAB species. 6](#_Toc107933262)

[Supplementary Figure 5. Median BGC count and proportion in SAGs. 7](#_Toc107933263)

[Supplementary Figure 6. Comparison of BGC proportion and counts in LAB. 8](#_Toc107933264)

[Supplementary Figure 7. Comparison of SM BGC capacity between LAB and non-LAB genera. 9](#_Toc107933265)

[Supplementary Figure 8. Distribution of 2,849 GCFs. 10](#_Toc107933266)

[Supplementary Figure 10. Domain distribution of 88 cross-genus RiPP-like GCFs. 12](#_Toc107933267)

[Supplementary Figure 11. BGCs are prevalent in six body sites. 13](#_Toc107933268)

[Supplementary Figure 12. Distribution of reference BGCs with different activities in training data. 14](#_Toc107933269)

[Supplementary Figure 13. Performances of four classifiers in determining activities of BGC-encoding compounds. 15](#_Toc107933270)

[Supplementary Figure 14. Profile of predicted compound activities of BGCs in LAB genera. 16](#_Toc107933271)

[Supplementary Figure 15. The predicted activity of cytotoxic and antifungal. 17](#_Toc107933272)

[Supplementary Figure 16. The sequence similarity network of precursor peptides reveals the huge diversity of putative class II bacteriocins. 18](#_Toc107933273)

[Supplementary Figure 17. The variable prevalence of class II bacteriocins in six body sites. 19](#_Toc107933274)

[Supplementary Figure 18. BGCs harboring precursors of cluster_467 and cluster_468. 20](#_Toc107933275)

[Supplementary Figure 19. HR-LCMS analysis of synthesized peptides. 21](#_Toc107933276)

**
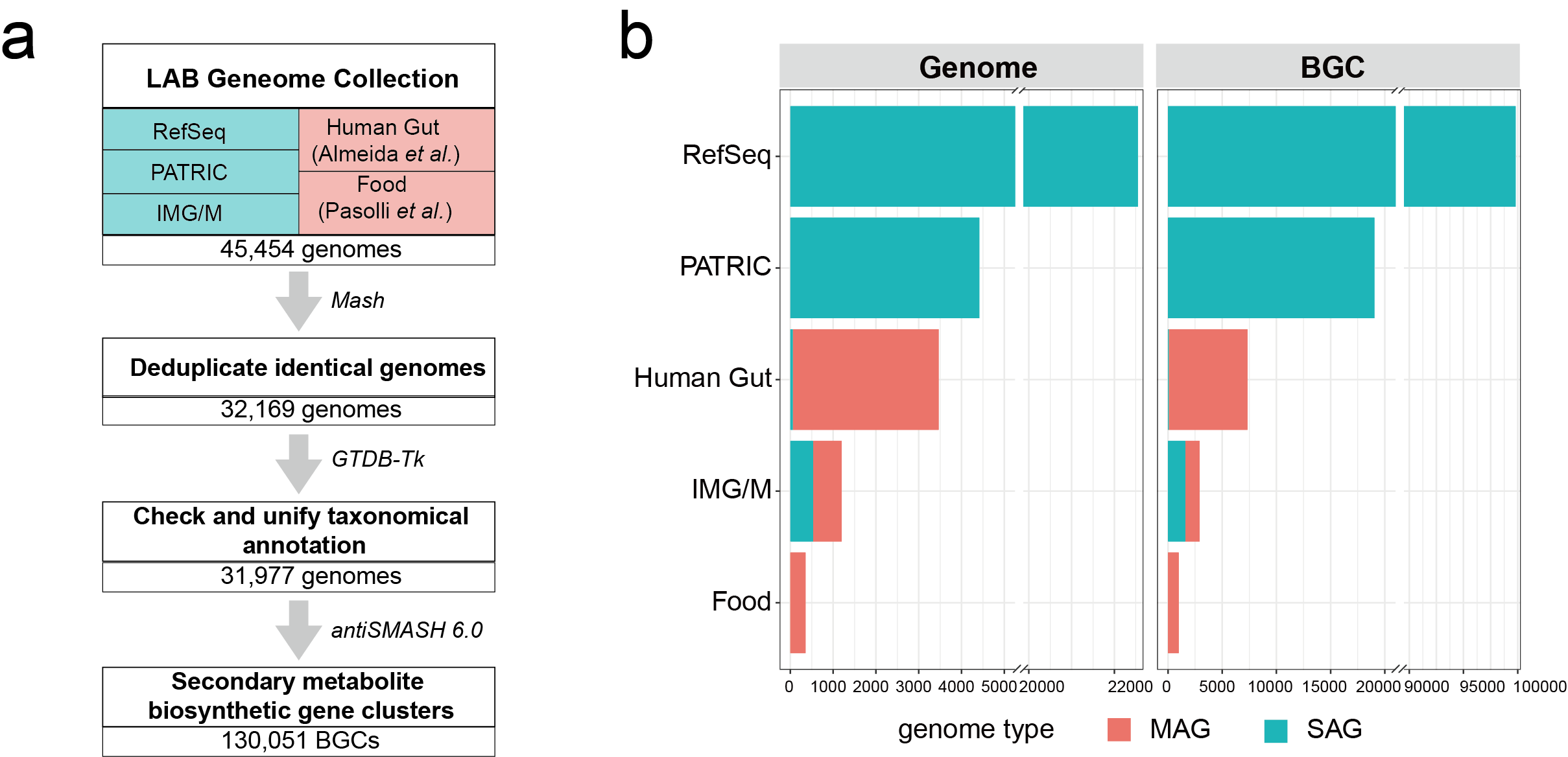
**

Supplementary Figure 1. Overview of the data processing. **a**, A flowchart showing the data collection and BGC annotation. LAB genomes were gathered from RefSeq, PATRIC, and IMG/M databases as well as two previous studies focusing on the human gut and food. **b**, Bar plot showing the number of LAB genomes and BGCs annotated. From 30,718 LAB genomes, a total of 130,051 BGCs were identified, with 120,501 from SAGs and 9,550 from MAGs.


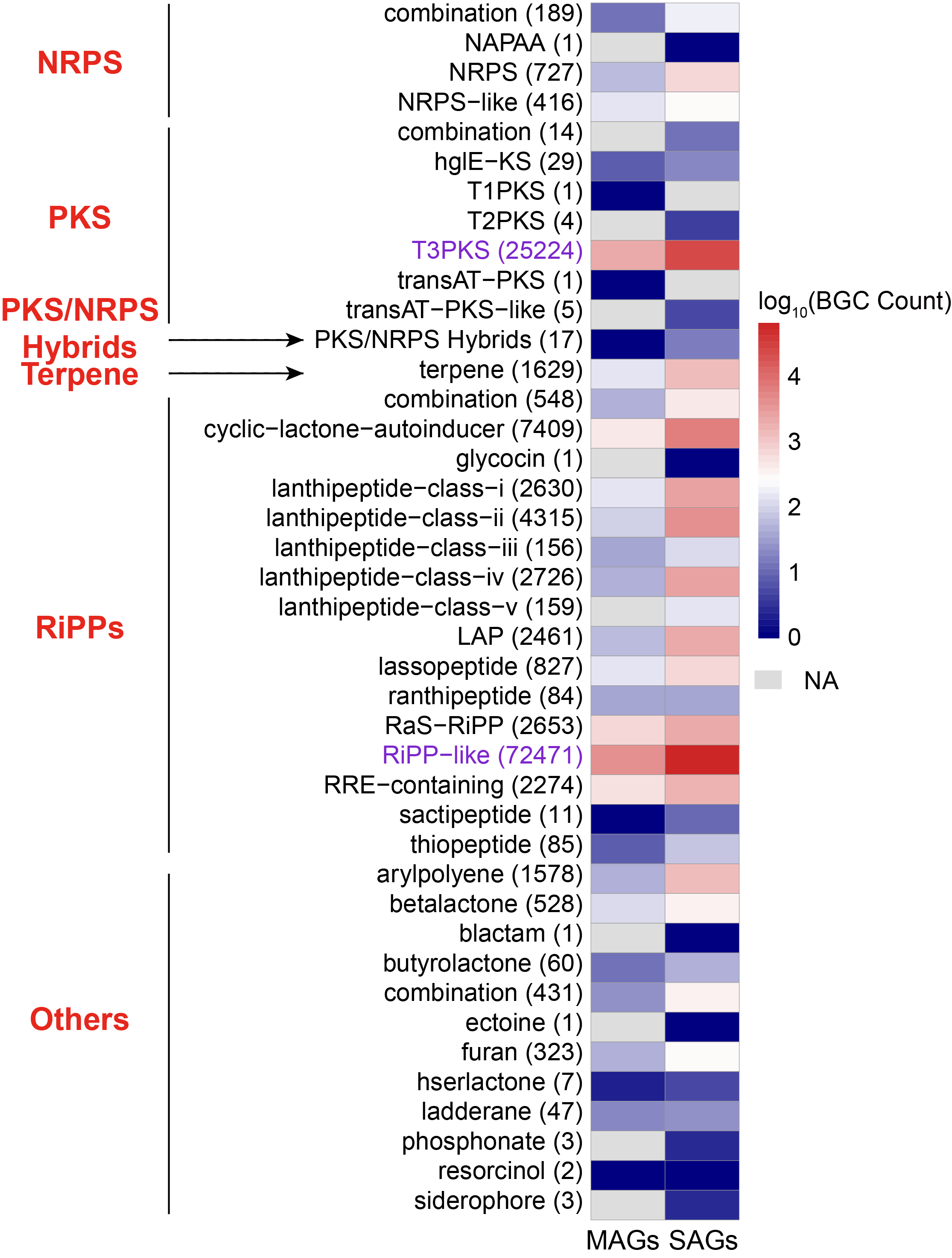


Supplementary Figure 2. Number of BGCs identified from MAGs and SAGs. The secondary metabolites of BGCs are annotated using antiSMASH. Those BGCs can be grouped into different classes (in red) which are applied in BiG-SCAPE (https://git.wageningenur.nl/medema-group/BiG-SCAPE/-/wikis/BiG-SCAPE%20classes). The numbers in brackets are the sum counts of BGCs from MAGs and SAGs, for each BGC class. The two most abundant BGCs, T3PKS, and RiPP-like, are highlighted in purple.

**
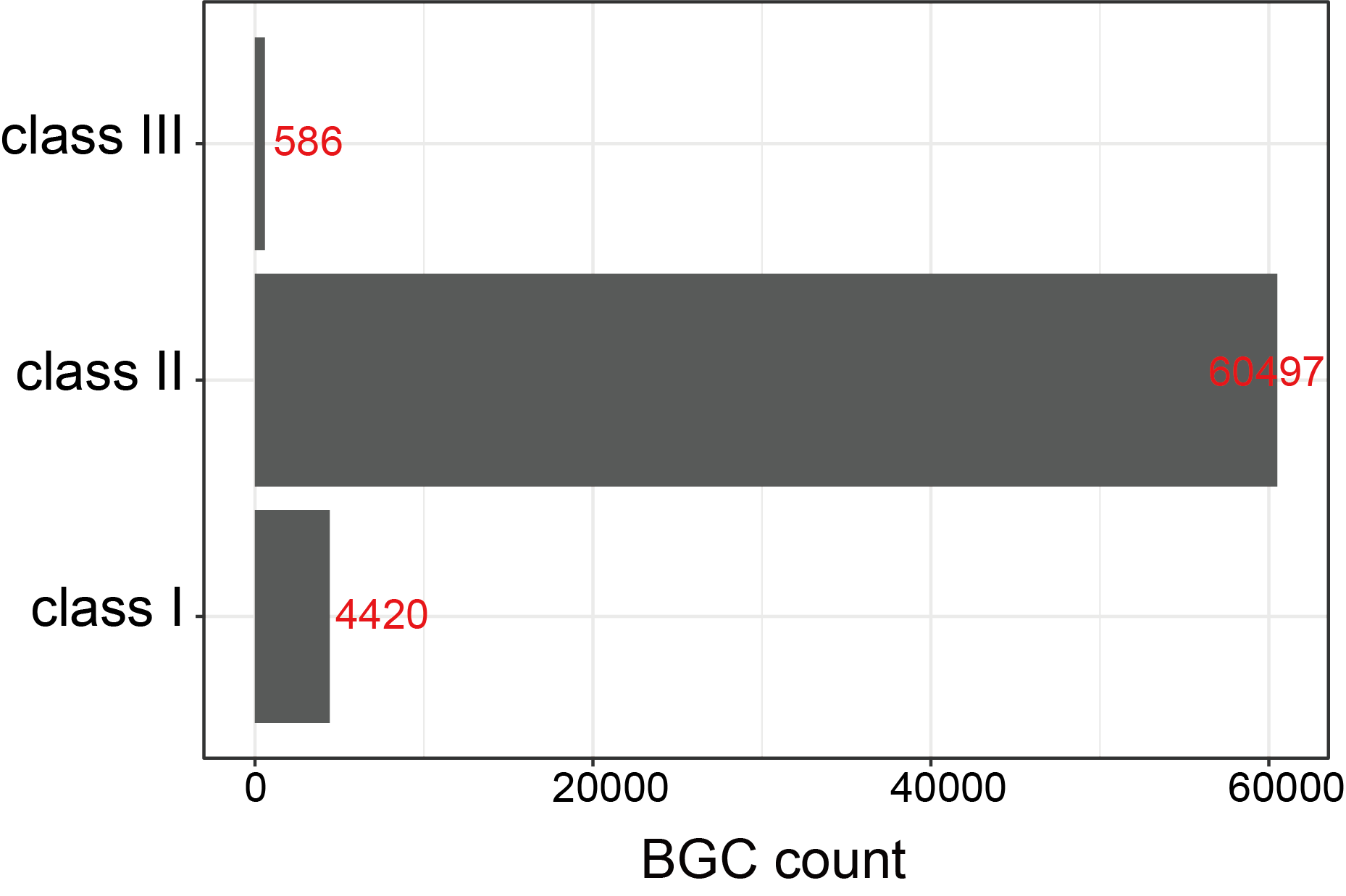
**

Supplementary Figure 3. The number of bacteriocins identified from 72,471 RiPP-like BGCs. As antiSMASH will annotate partial class I, class II, and class III bacteriocins as RiPP-like, we then used BiG-SLiCE to extract bacteriocin biosynthesis-related domains (provided in Supplementary Table 4). We further identified 4,420 class I bacteriocins, 60,497 class II bacteriocins (46.5%), and 586 class III bacteriocins (0.5%) from RiPP-like BGCs. It should be noted that other RiPPs such as lanthipeptides, thiopeptides, and lassopeptides could be classed into class I bacteriocins if they were active against bacteria.

**
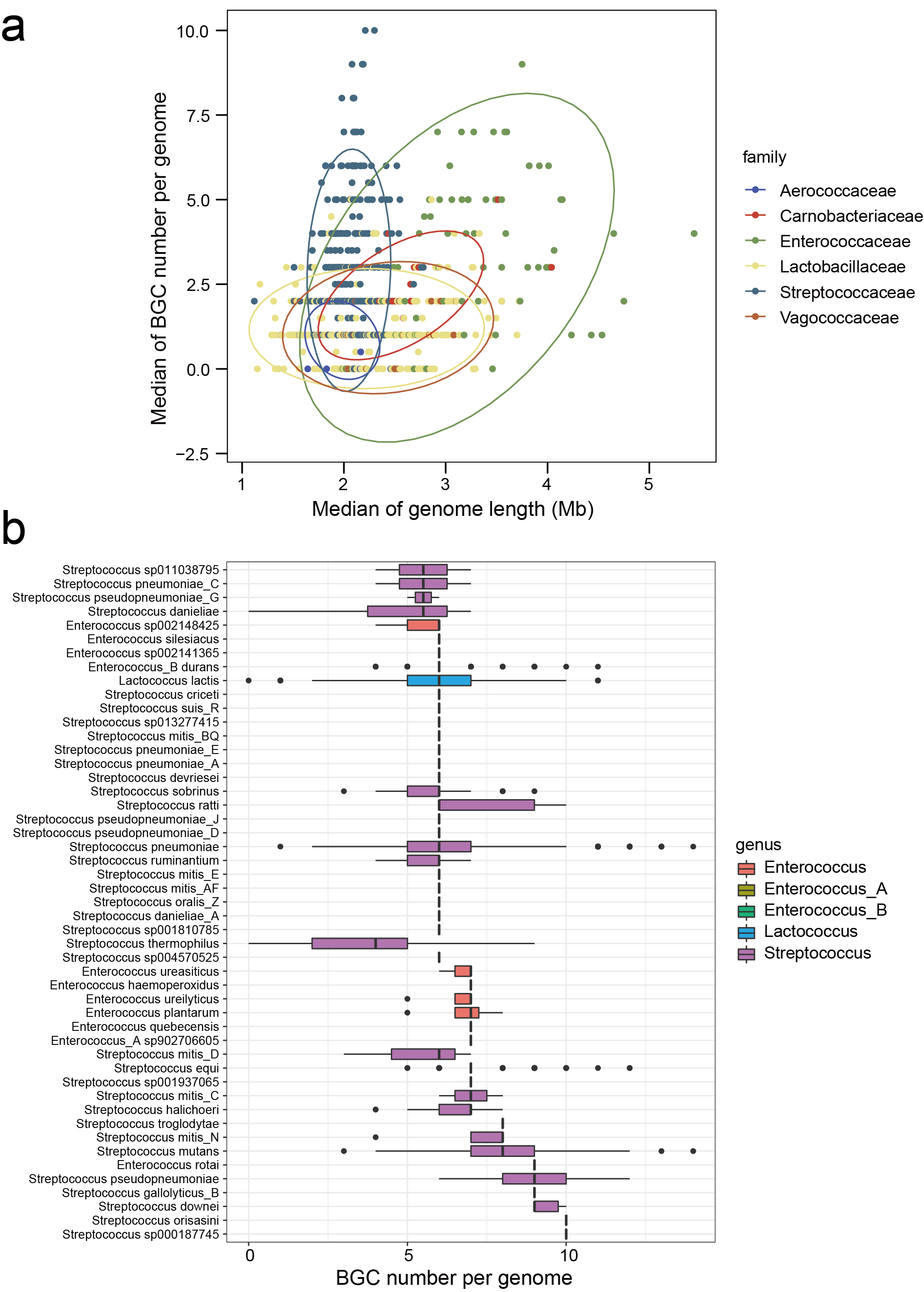
**

Supplementary Figure 4. Biosynthetic potential in LAB species. **a**, Scatterplot showing the profile of BGC number per genome within LAB species, colored according to the family level. Each dot stands for one species. Despite being small in genome size, the family Streptococcaceae generally harbored more abundant BGCs than other families. **b**, The distribution of BGC number per genome within 49 species with a median BGC number per genome of >5. Among them, 37 of 49 are *Streptococcus,* while 9 are *Enterococcus*.

**
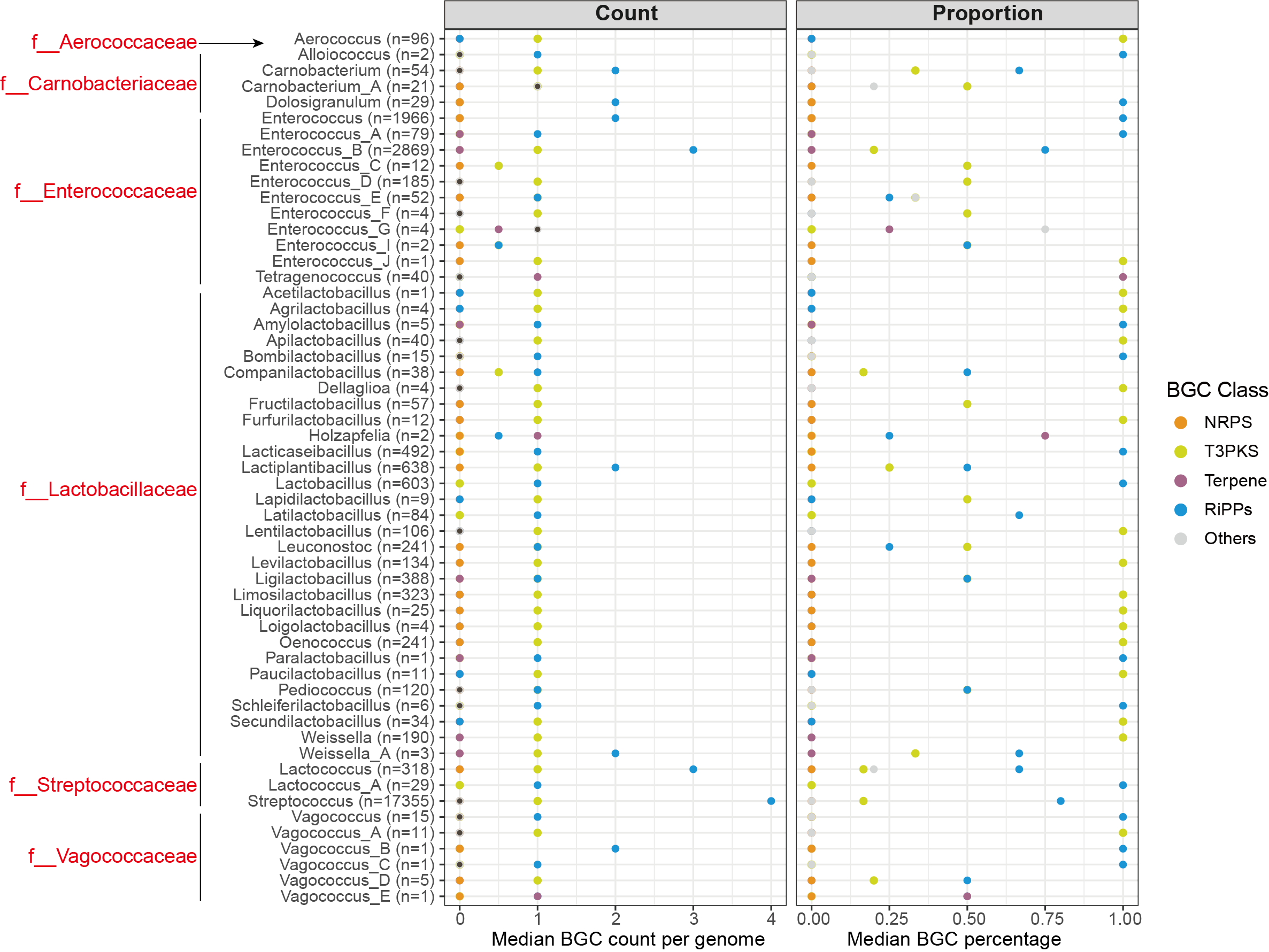
**

Supplementary Figure 5. Median BGC count and proportion in SAGs. The numbers in brackets are counts of BGC-containing SAGs for each genus.

**
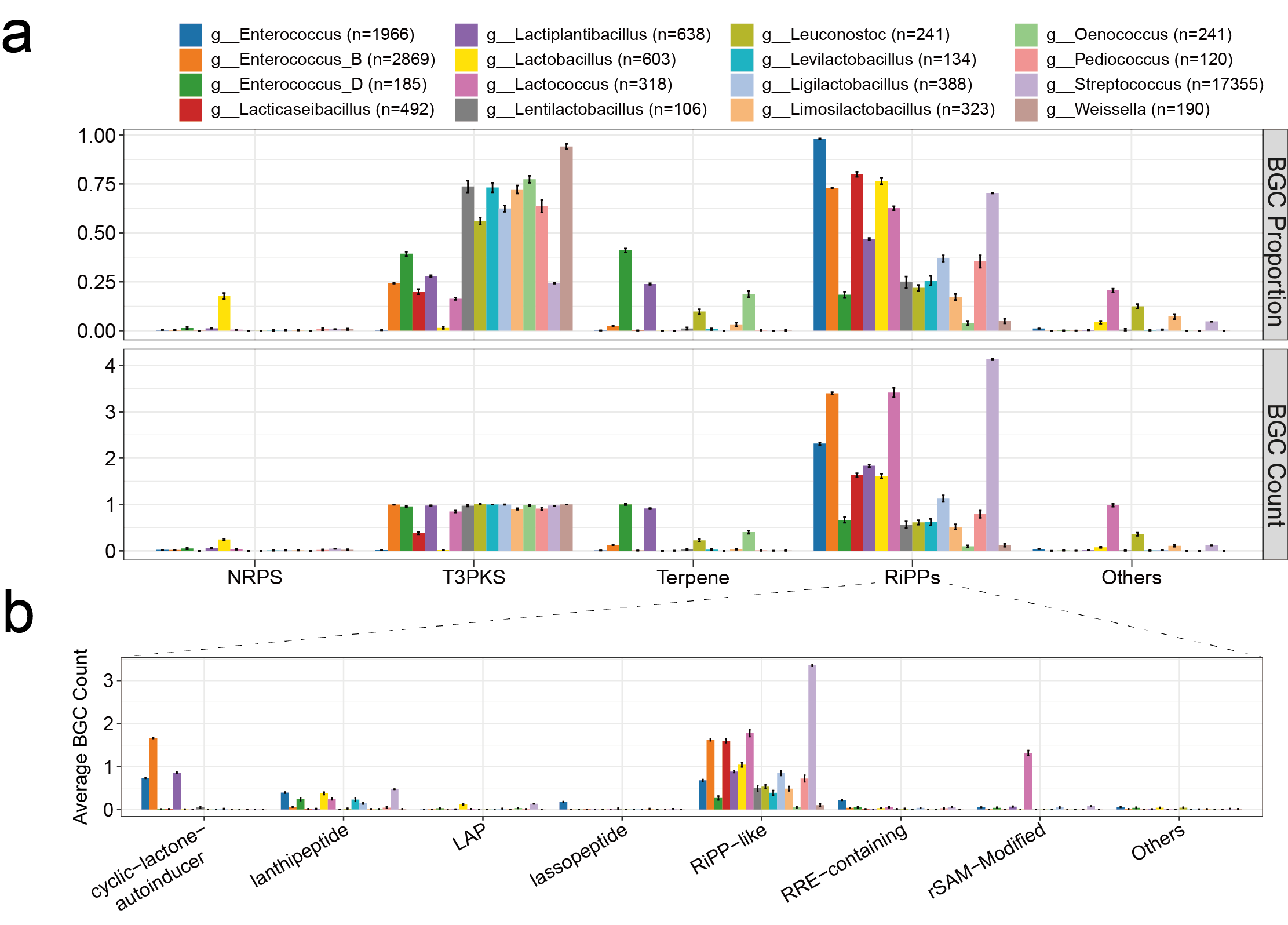
**

Supplementary Figure 6. Comparison of BGC proportion and counts in LAB. **a**, Bar plot showing the average BGC percentage (upper) and average BGC count per genome (lower) in 16 genera that comprise over 100 SAGs. To reduce the sampling bise, the average BGC percentage/count was calculated from half of the genomes of this genus, with 1,000 replicates of sampling. The numbers in brackets indicate the number of SAGs for each genus. **b**, The abundance of distinct RiPPs in 16 genera. rSAM-Modified RiPPs consist of RaS-RiPP, ranthipeptide, and sactipeptide. Other RiPPs or combinations of different RiPPs types are grouped into “Others”. Data are mean ± standard deviation.


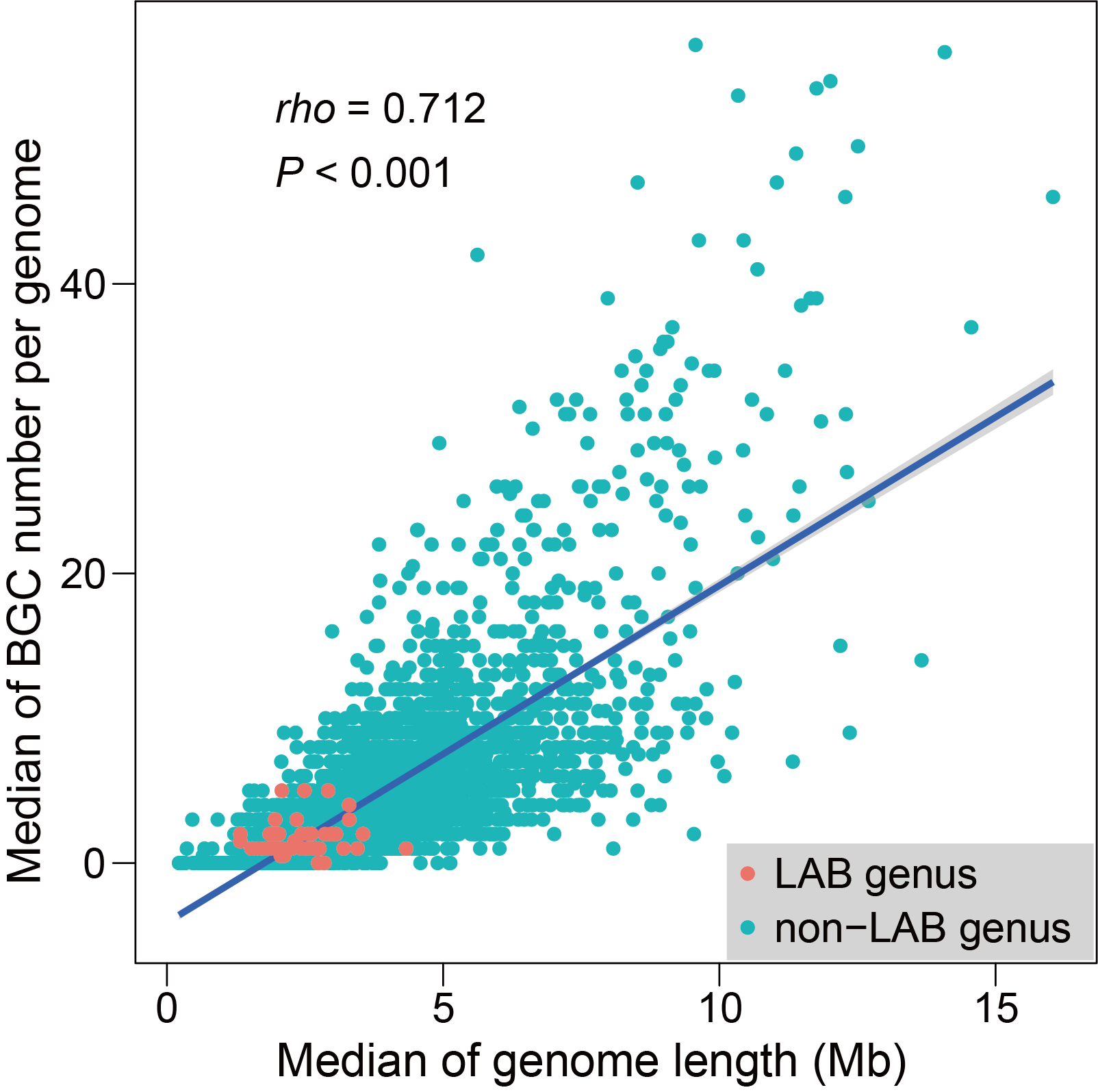


Supplementary Figure 7. Comparison of SM BGC capacity between LAB and non-LAB genera. Each dot represents one genus, with 56 LAB genera and 3,805 non-LAB genera. Spearman’s rank correlation was carried out, and significance was verified. The grey shade shows a 95% confidence interval.

**
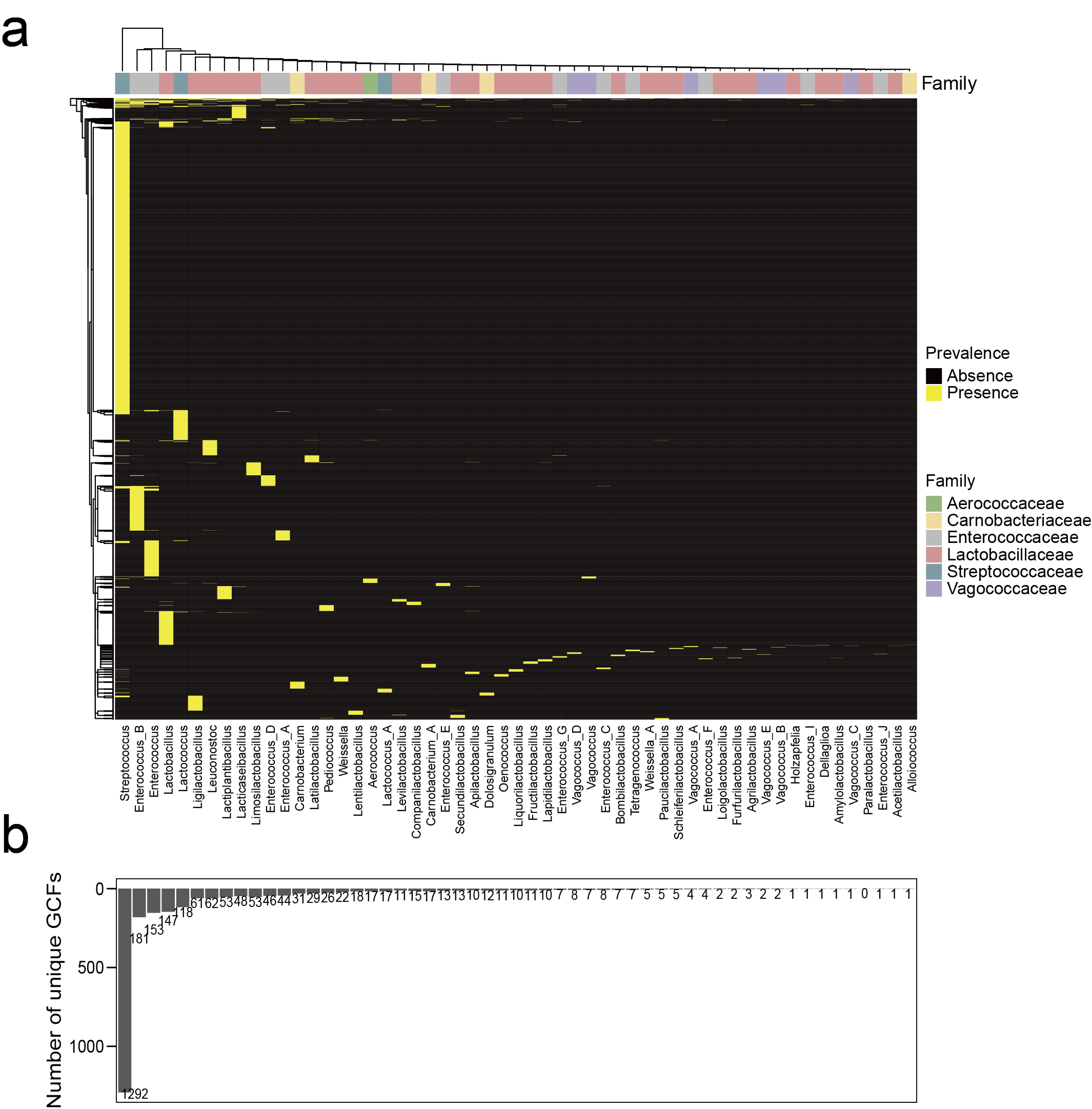
**

Supplementary Figure 8. Distribution of 2,849 GCFs. **a**, Distribution of 2,849 GCF in different genera. Each row represents one GCF. The genera of one family do not cluster together, indicating that the GCF between genera varies considerably and does not exhibit phylogeny-relatedness at the genus level. **b**, Number of unique GCFs in each genus.


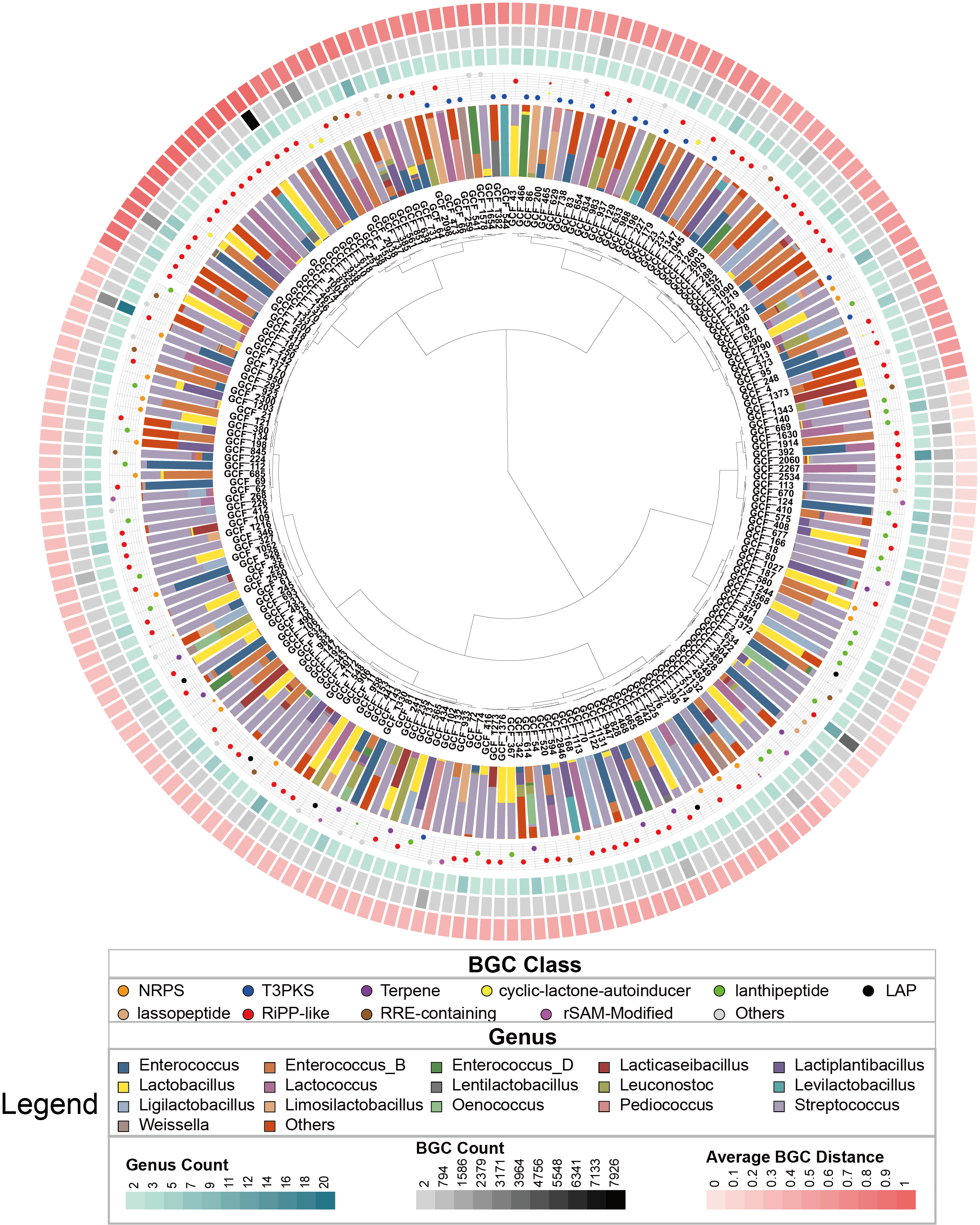
Supplementary Figure 9. Diversity of 212 cross-genus GCFs. Layers from inner to outer are (1) hierarchical clustering of 212 genera present in >1 genera, based on average BGC distance to known BGCs (experimentally validated BGCs in MIBiG database); (2) the proportion of distinct genera; (3) the percentage of BGC classes in each GCF. Point size is proportionate to the percentage; (4) the number of genera in which the GCF distributes; (5) BGC count in each GCF; (6) average BGC distance to MiBiG BGCs. Among 212 GCFs, the number of GCFs dominated by different classes of BGCs is as follows: NRPS, 17; T3PKS, 19; terpene, 9; cyclic-lactone-autoinducer, 4; lanthipeptide, 4; LAP, 5; lassopeptide, 4; RiPP-like, 88; RRE-containing, 10; rSAM-Modified, 5.

**
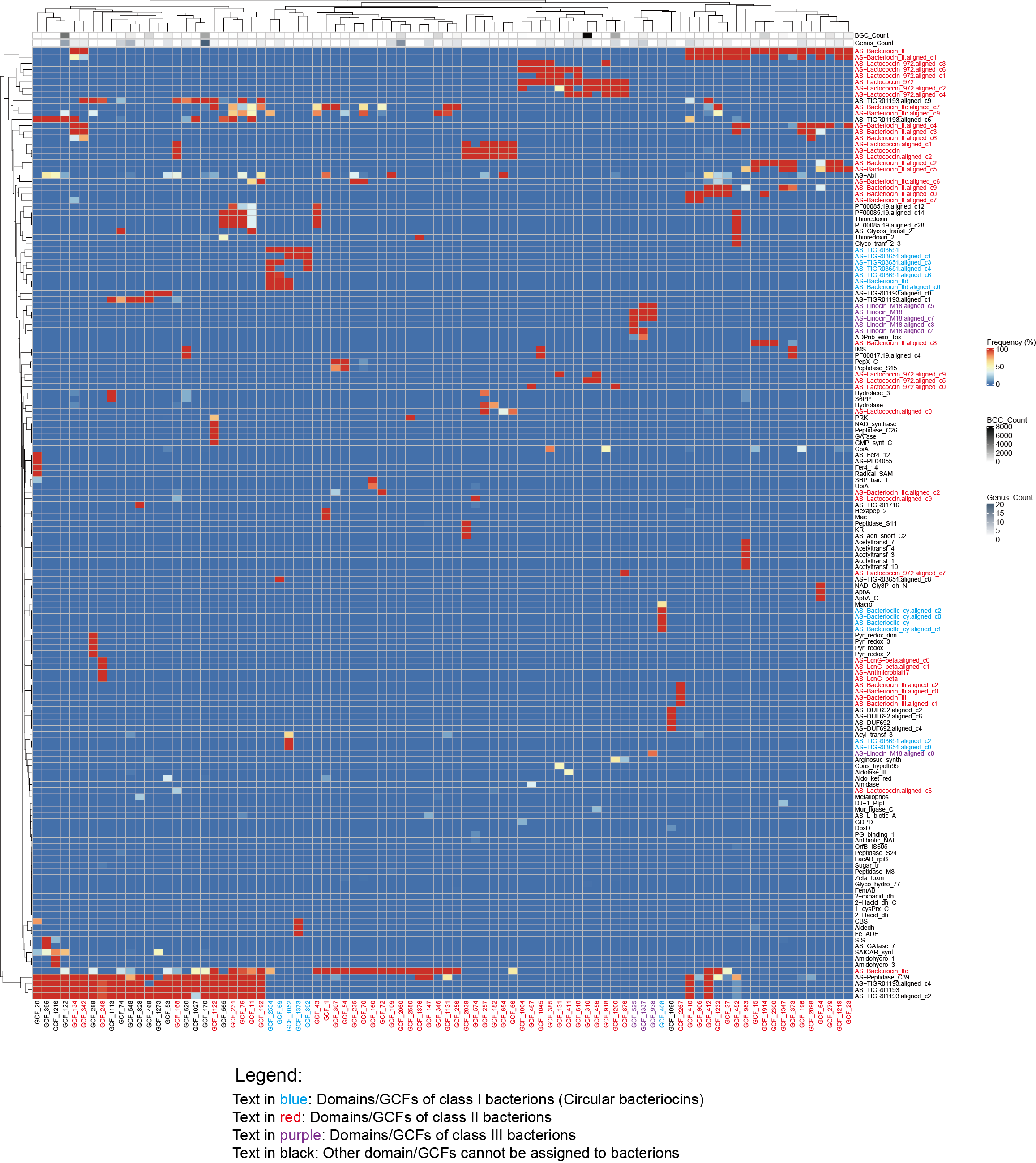
**

Supplementary Figure 10. Domain distribution of 88 cross-genus RiPP-like GCFs. The heatmap shows the percentage of predominant BGC feature domains (extracted by BiG-SLiCE) in 88 RiPP-like GCFs, most of which are class II bacteriocins (62 GCFs). For each GCF, the top five prevalent domains (with a high proportion of occurrence) are fetched out, and all prevalent domains retrieved are shown on the heatmap. Characteristic domains of class I, class II, and class III bacteriocin are highlighted in blue, red, and purple.

**
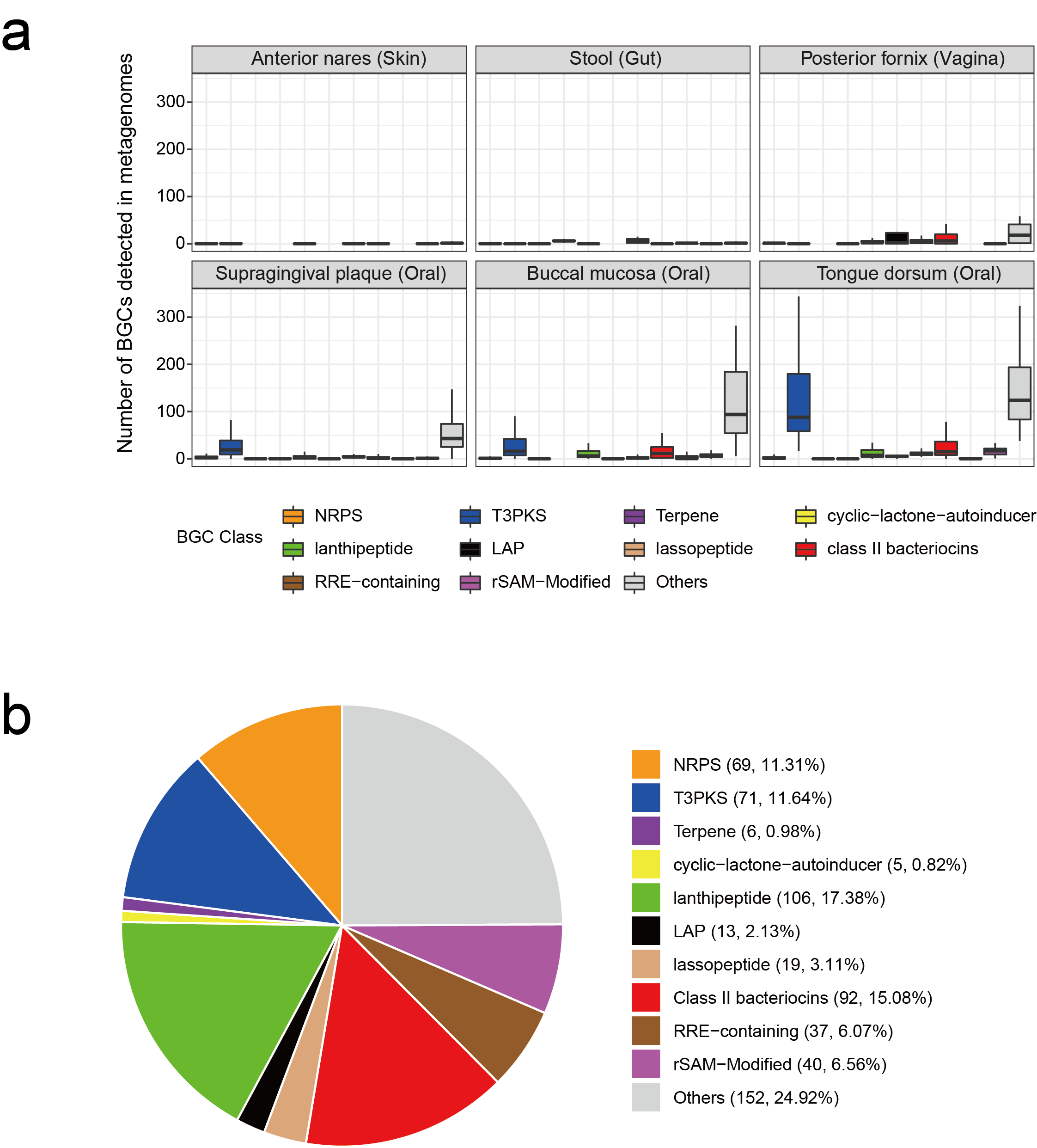
**

Supplementary Figure 11. BGCs are prevalent in six body sites. **a,** The box plots show the number of BGCs detected in metagenomes of six body sites. BGCs are stratified as per different classes. BGCs that harbor class II bacteriocins-related domains (shown in Supplementary Figures 9, 10) are grouped into class II bacteriocins. b, The prevalence of GCFs detected in six body sites. The numbers in the bracket are the number of GCFs and the corresponding percentage.

**
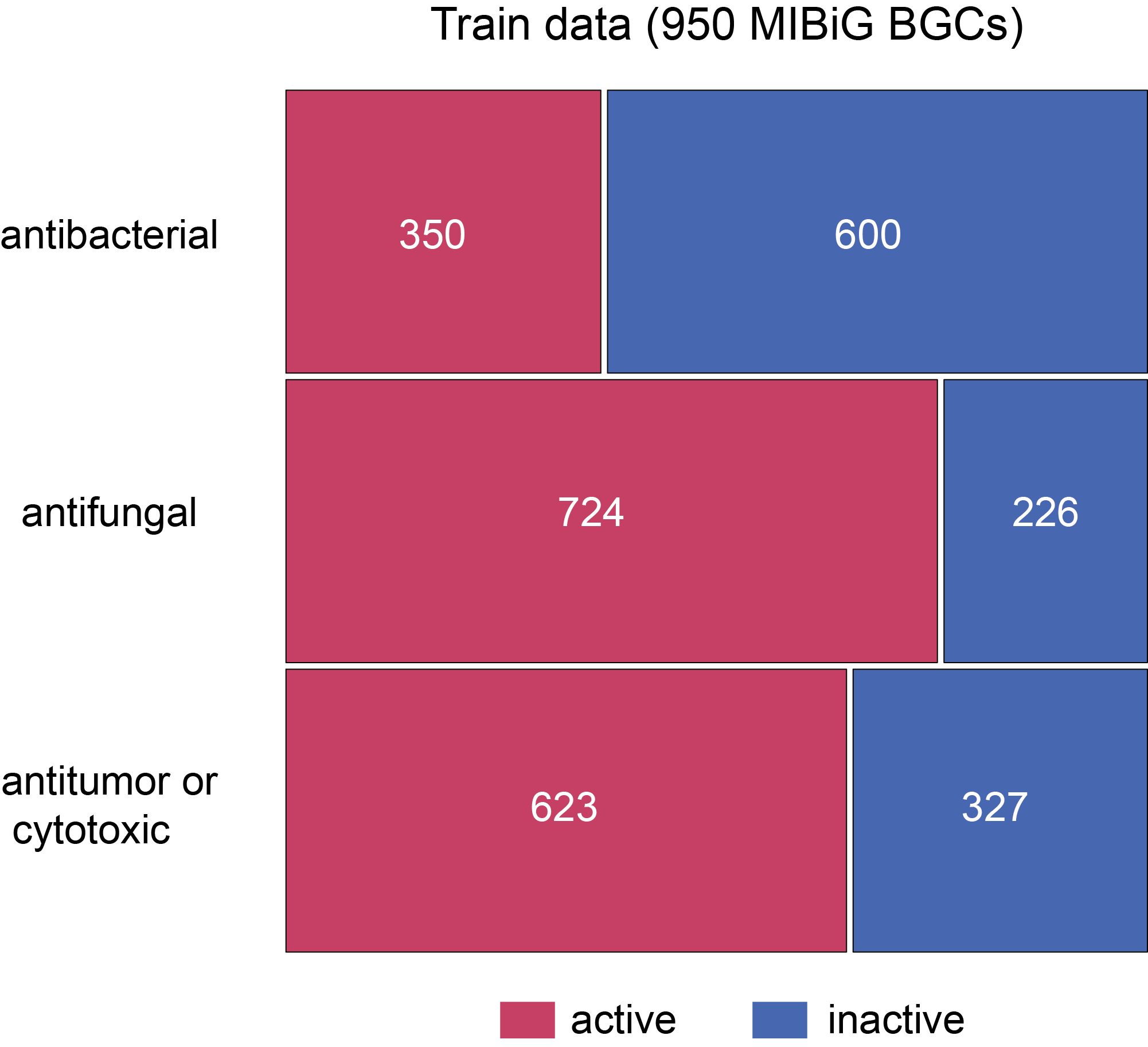
**

Supplementary Figure 12. Distribution of reference BGCs with different activities in training data. 950 MIBiG BGCs with known functions were used as the train data set. The number denotes the BGC counts.

**
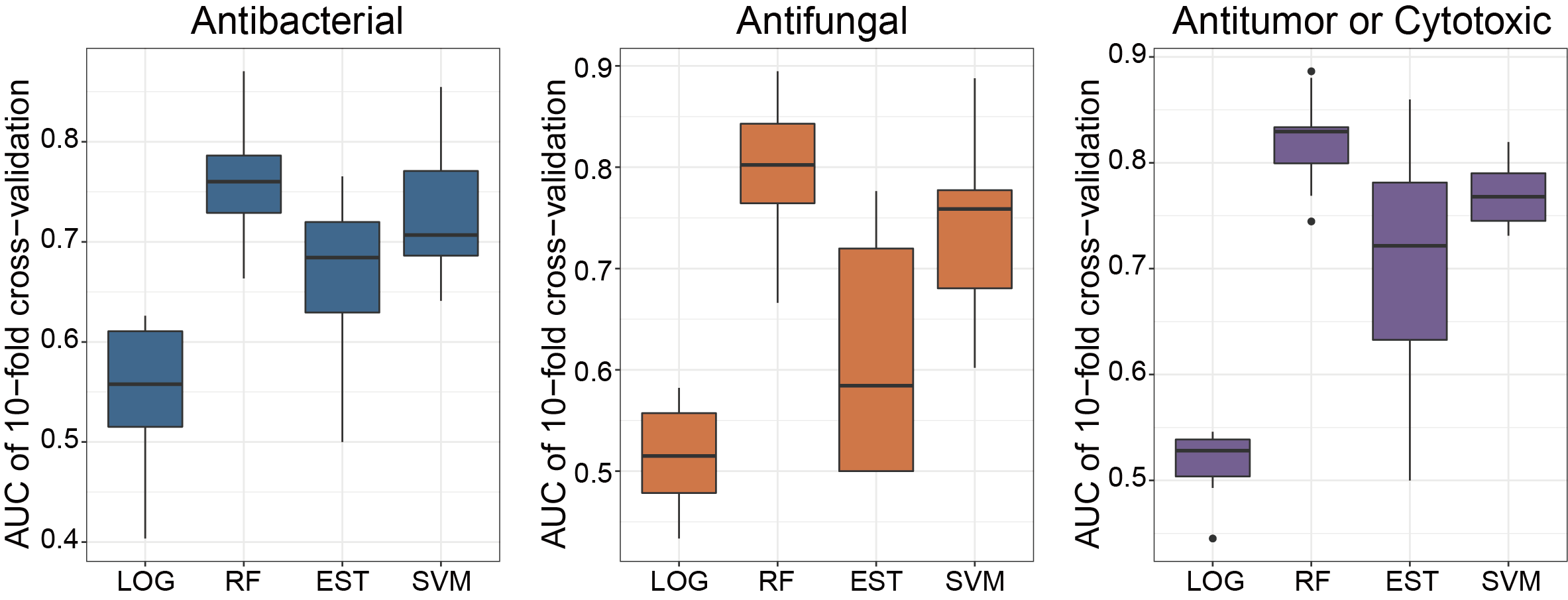
**

Supplementary Figure 13. Performances of four classifiers in determining activities of BGC-encoding compounds. The performances were quantified with index AUC [area under the ROC curve (receiver operating characteristic curve)], using 10-fold cross-validation. LOG, logistic regression; RF, random forest; EST, elastic net regression; SVM, support vector machines.

**
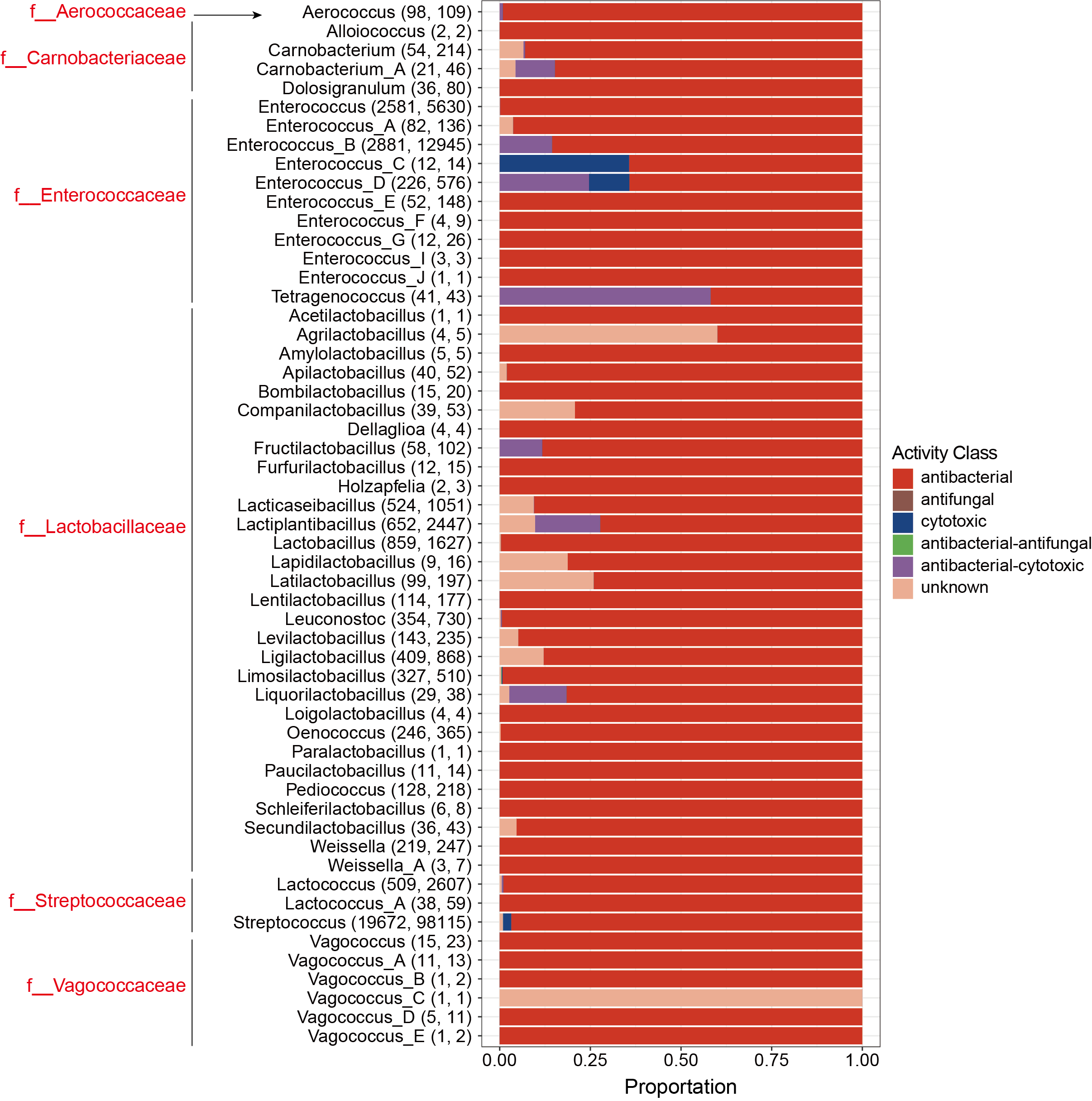
**

Supplementary Figure 14. Profile of predicted compound activities of BGCs in LAB genera. The numbers in the bracket are genome count and total BGC count, respectively.


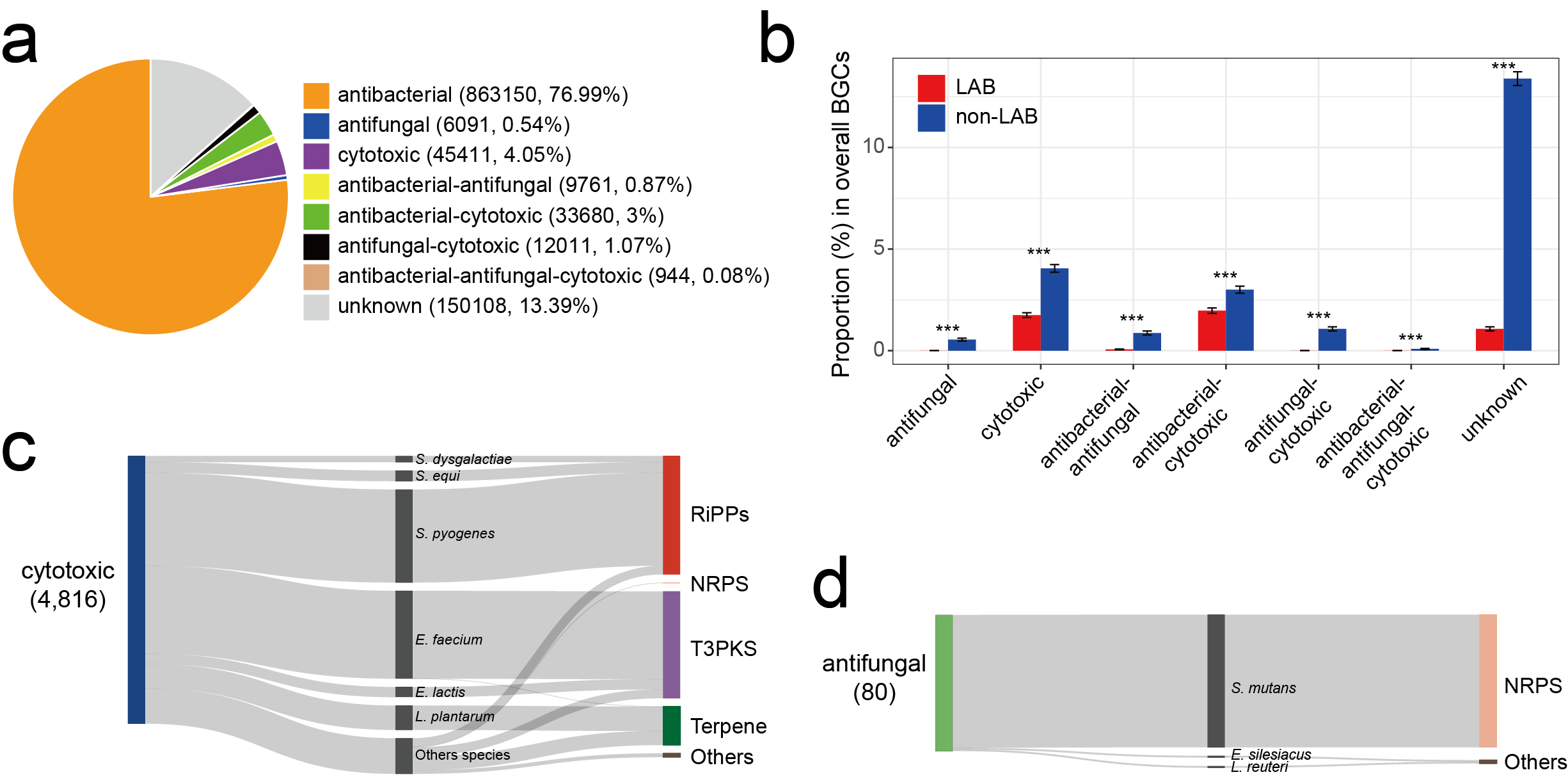


Supplementary Figure 15. The predicted activity of BGCs from non-LAB and LAB genomes. a The Proportion of different activities predicted from 1,121,156 BGCs of non-LAB. **b,** Proportion of SMs with different activities encoded by BGCs from LAB and non-LAB. We randomly selected 10,000 BGCs of LAB or non-LAB 1,000 times, which were used to compute the proportion. Data are mean ± standard deviation. *P* value was given by Wilcoxon rank-sum test (two sided), with “***” denoting *P* < 0.001. **c, d** Sankey diagrams show the LAB-derived BGCs encoding SMs with the predicted activity of cytotoxic (**c**) and antifungal (**d**). The number shown in brackets refers to the BGC count. The full names of bacteria are as follows: *S. dysgalactiae*, *Streptococcus dysgalactiae*; *S. equi*, *Streptococcus equi*; *S. pyogenes*, *Streptococcus pyogenes*; *E. faecium*, *Enterococcus faecium*; *E. lactis*, *Enterococcus lactis*; *L. plantarum*, *Lactiplantibacillus plantarum*; *S. mutans*, *Streptococcus mutans*; *E. silesiacus,* *Enterococcus silesiacus*; *L. reuteri*, *Limosilactobacillus reuteri*.

**
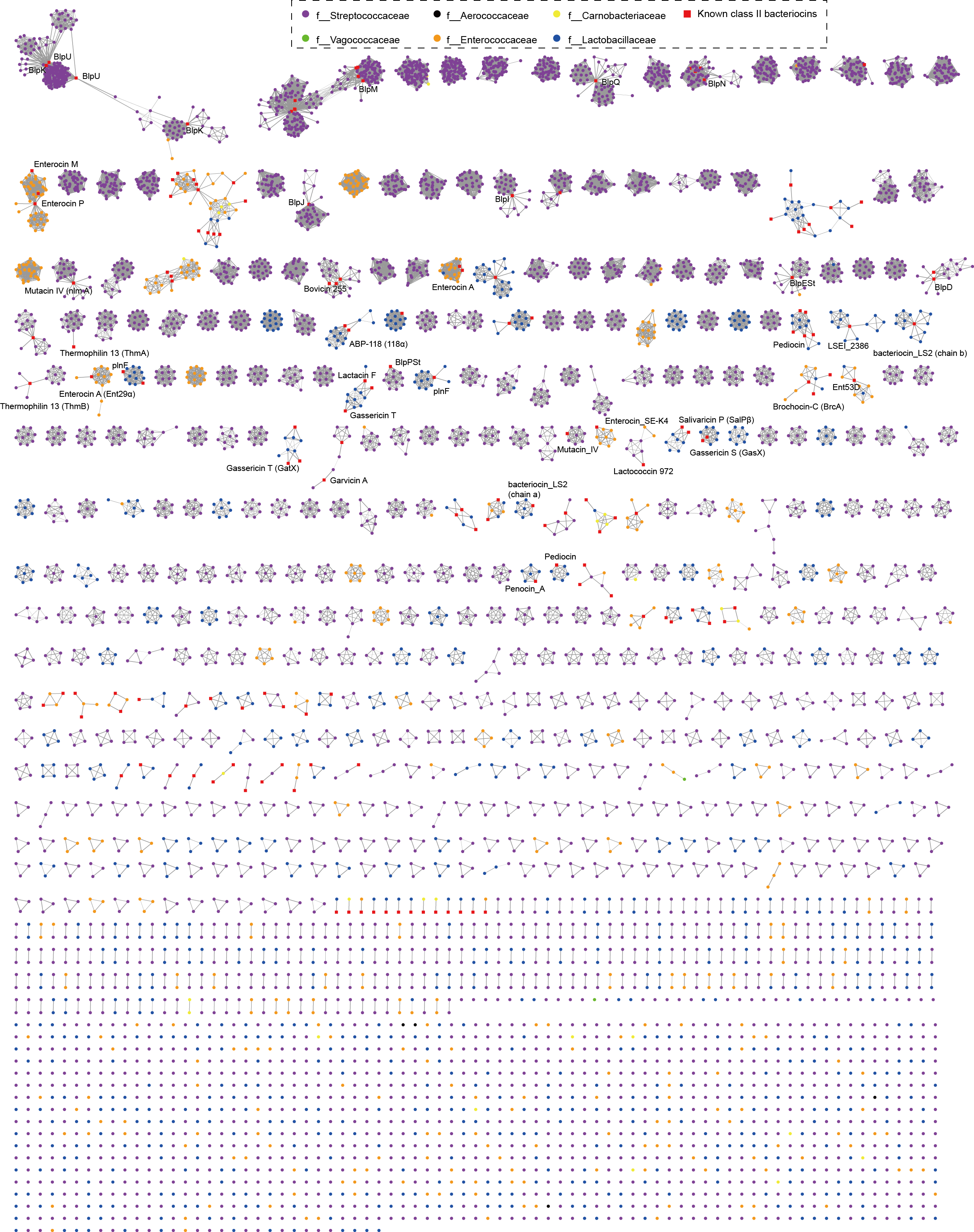
**

Supplementary Figure 16. The sequence similarity network of precursor peptides reveals the huge diversity of putative class II bacteriocins. The 187,649 precursor sequences were de-duplicated to 6,516 sequences, shown in the network. Within each cluster, two sequences with identity > 50% are connected with a line. Precursor sequence and known class II bacteriocins are connected only if the identity > 90% and coverage > 95%. The line width is proportionate to the sequence identity. Each dot represents one precursor sequence, which is further colored according to the LAB family. The vast majority of precursors did not cluster with the known bacteriocins, demonstrating their potential for discovering novel bacteriocins.

**
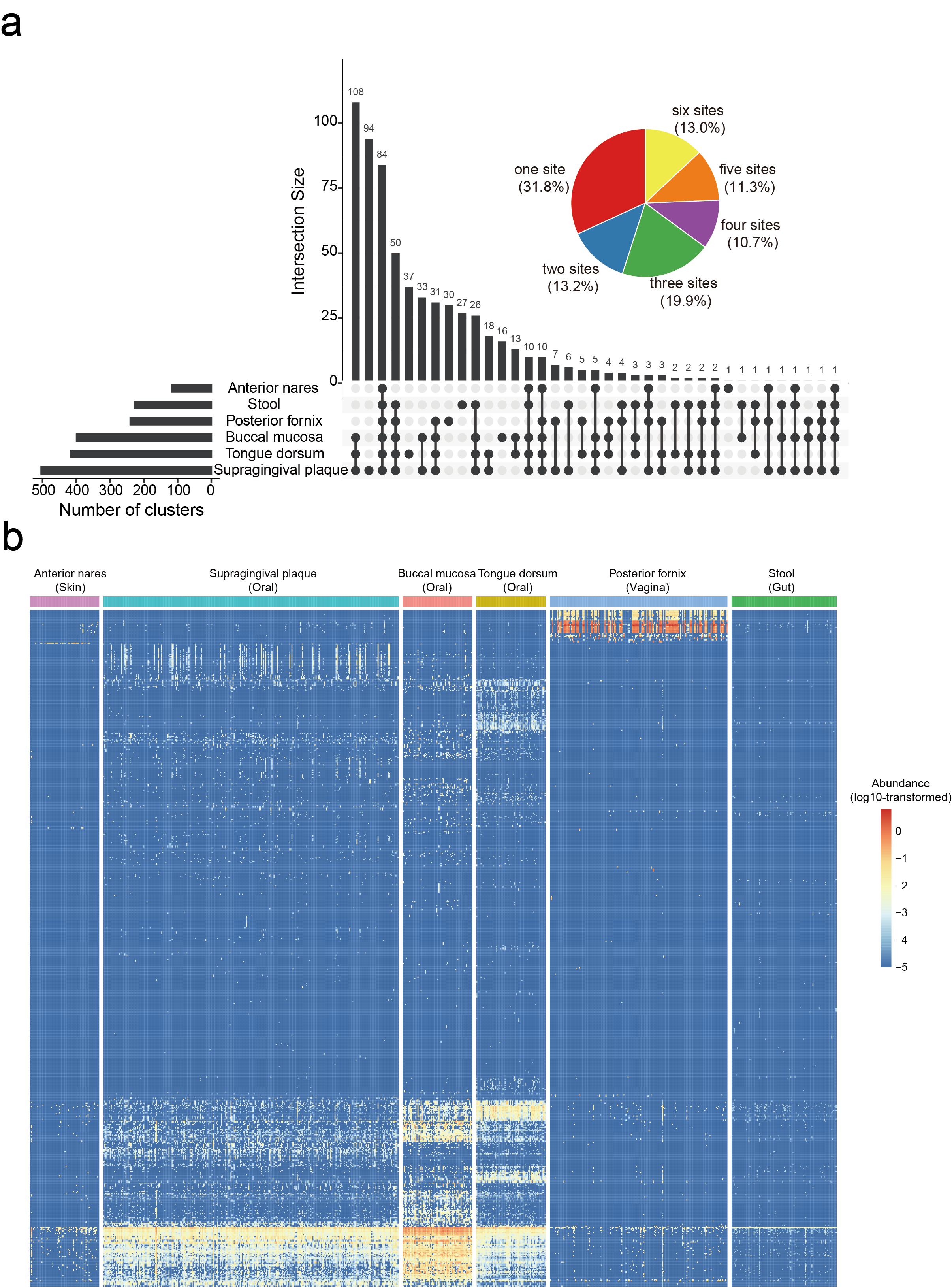
**

Supplementary Figure 17. The variable prevalence of class II bacteriocins in six body sites. **a**, The intersection of clusters detected in different body sites. The bar plot on the left shows the cluster count in each body site; the bar plot on the top refers to the number of clusters of each intersection. Connecting lines are drawn if an intersection is present in more than one site. The pie chart shows the proportion of 644 clusters detected in different body sites. The corresponding percentages are shown in the brackets. **b**, The heat map shows the profile of 644 clusters in human metagenomes. The heat map reveals different predominant clusters in the vagina and oral cavity. Together, these results revealed the niche specificity of class II bacteriocins in the human microbiome.

**
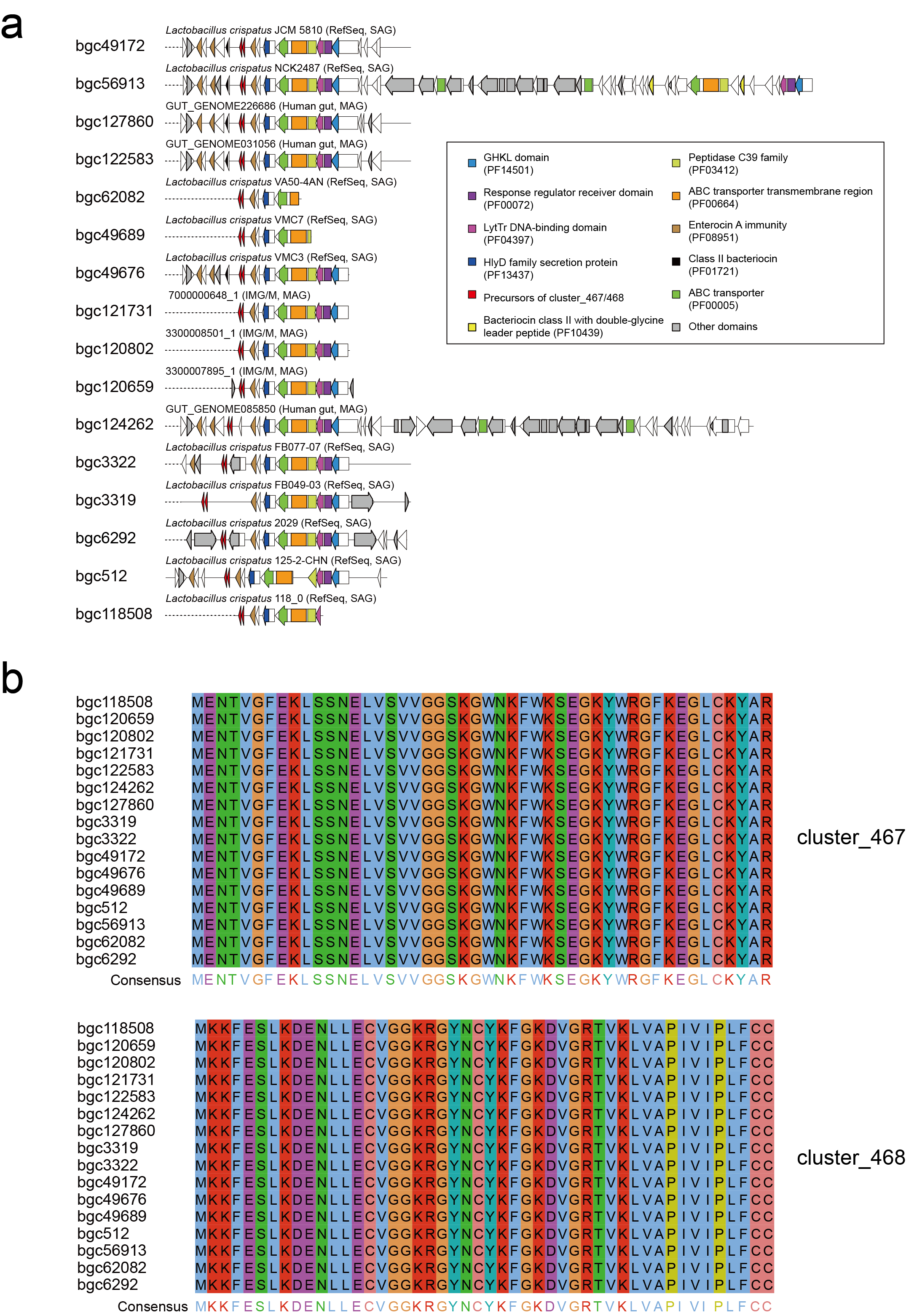
**

Supplementary Figure 18. BGCs harboring precursors of cluster_467 and cluster_468. **a**, Gene organizations of 18 BGCs containing putative precursors belonging to cluter_467 and cluster_468. BiG-SCAPE detected the biosynthetic related domains. Ten BGCs from isolates deposited in the RefSeq database, three BGCs from human gut metagenomes, and three BGC from metagenomes deposited in IMG/M database were labeled on the top of the BGC architecture. **b**, Precursor peptides corresponding to the 16 BGCs shown in (**a**).

**
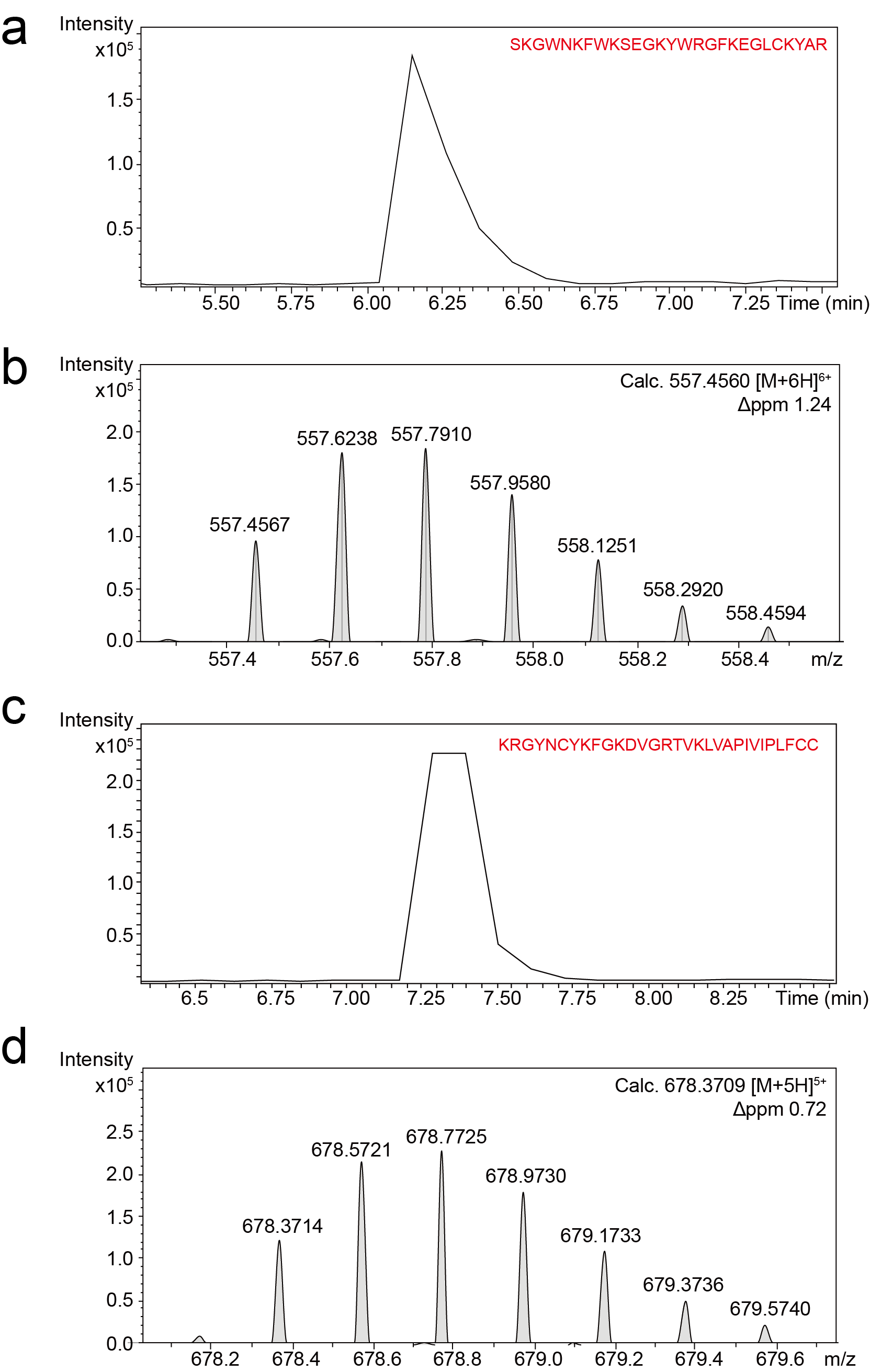
**

Supplementary Figure 19. HR-LCMS analysis of synthesized peptides. HR-LCMS analysis of two core peptides is shown here, including the retention time (**a, c**) and their MS1 spectra (**b, d**) for cluster_467 (**a, b**) and cluster_468 (**c, d**).
